# Supplementary figures and images for: Food Image Segmentation Using Multi-Modal Imaging Sensors with Color and Thermal Data (part 2 of 2)
Source: Sensors (Basel). 2023 Jan 4;23(2):560. doi: 10.3390/s23020560 (PMC9860575; doi:10.3390/s23020560)

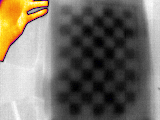

Supplement: Supplementary file 1 [file sensors-23-00560-s001.zip › Combined Data/Calibration/Calibration thermal/rgb53.png]

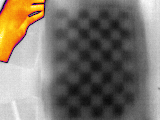

Supplement: Supplementary file 1 [file sensors-23-00560-s001.zip › Combined Data/Calibration/Calibration thermal/rgb54.png]

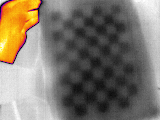

Supplement: Supplementary file 1 [file sensors-23-00560-s001.zip › Combined Data/Calibration/Calibration thermal/rgb55.png]

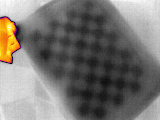

Supplement: Supplementary file 1 [file sensors-23-00560-s001.zip › Combined Data/Calibration/Calibration thermal/rgb56.png]

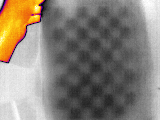

Supplement: Supplementary file 1 [file sensors-23-00560-s001.zip › Combined Data/Calibration/Calibration thermal/rgb57.png]

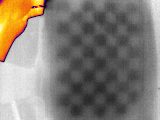

Supplement: Supplementary file 1 [file sensors-23-00560-s001.zip › Combined Data/Calibration/Calibration thermal/rgb58.png]

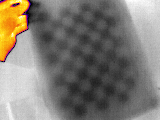

Supplement: Supplementary file 1 [file sensors-23-00560-s001.zip › Combined Data/Calibration/Calibration thermal/rgb59.png]

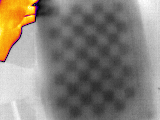

Supplement: Supplementary file 1 [file sensors-23-00560-s001.zip › Combined Data/Calibration/Calibration thermal/rgb60.png]

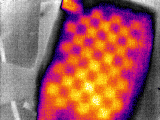

Supplement: Supplementary file 1 [file sensors-23-00560-s001.zip › Combined Data/Calibration/Calibration thermal/rgb61.png]

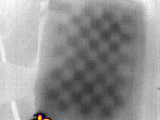

Supplement: Supplementary file 1 [file sensors-23-00560-s001.zip › Combined Data/Calibration/Calibration thermal/rgb62.png]

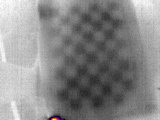

Supplement: Supplementary file 1 [file sensors-23-00560-s001.zip › Combined Data/Calibration/Calibration thermal/rgb63.png]

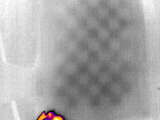

Supplement: Supplementary file 1 [file sensors-23-00560-s001.zip › Combined Data/Calibration/Calibration thermal/rgb64.png]

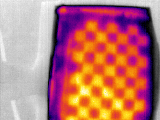

Supplement: Supplementary file 1 [file sensors-23-00560-s001.zip › Combined Data/Calibration/Calibration thermal/rgb65.png]

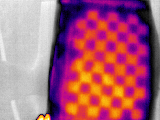

Supplement: Supplementary file 1 [file sensors-23-00560-s001.zip › Combined Data/Calibration/Calibration thermal/rgb66.png]

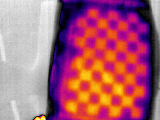

Supplement: Supplementary file 1 [file sensors-23-00560-s001.zip › Combined Data/Calibration/Calibration thermal/rgb67.png]

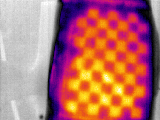

Supplement: Supplementary file 1 [file sensors-23-00560-s001.zip › Combined Data/Calibration/Calibration thermal/rgb68.png]

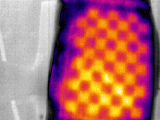

Supplement: Supplementary file 1 [file sensors-23-00560-s001.zip › Combined Data/Calibration/Calibration thermal/rgb69.png]

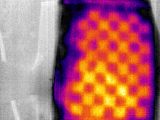

Supplement: Supplementary file 1 [file sensors-23-00560-s001.zip › Combined Data/Calibration/Calibration thermal/rgb70.png]

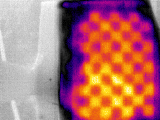

Supplement: Supplementary file 1 [file sensors-23-00560-s001.zip › Combined Data/Calibration/Calibration thermal/rgb71.png]

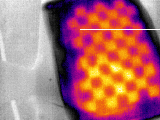

Supplement: Supplementary file 1 [file sensors-23-00560-s001.zip › Combined Data/Calibration/Calibration thermal/rgb72.png]

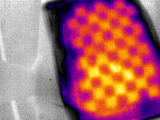

Supplement: Supplementary file 1 [file sensors-23-00560-s001.zip › Combined Data/Calibration/Calibration thermal/rgb73.png]

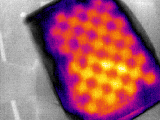

Supplement: Supplementary file 1 [file sensors-23-00560-s001.zip › Combined Data/Calibration/Calibration thermal/rgb74.png]

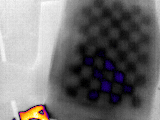

Supplement: Supplementary file 1 [file sensors-23-00560-s001.zip › Combined Data/Calibration/Calibration thermal/rgb75.png]

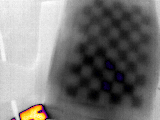

Supplement: Supplementary file 1 [file sensors-23-00560-s001.zip › Combined Data/Calibration/Calibration thermal/rgb76.png]

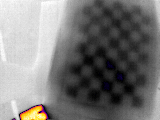

Supplement: Supplementary file 1 [file sensors-23-00560-s001.zip › Combined Data/Calibration/Calibration thermal/rgb77.png]

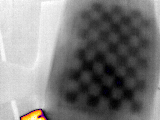

Supplement: Supplementary file 1 [file sensors-23-00560-s001.zip › Combined Data/Calibration/Calibration thermal/rgb78.png]

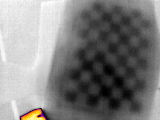

Supplement: Supplementary file 1 [file sensors-23-00560-s001.zip › Combined Data/Calibration/Calibration thermal/rgb79.png]

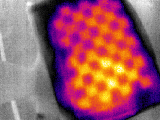

Supplement: Supplementary file 1 [file sensors-23-00560-s001.zip › Combined Data/Calibration/Calibration thermal/rgb80.png]

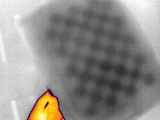

Supplement: Supplementary file 1 [file sensors-23-00560-s001.zip › Combined Data/Calibration/Calibration thermal/rgb81.png]

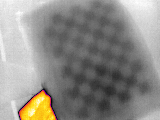

Supplement: Supplementary file 1 [file sensors-23-00560-s001.zip › Combined Data/Calibration/Calibration thermal/rgb82.png]

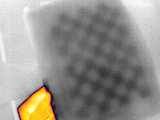

Supplement: Supplementary file 1 [file sensors-23-00560-s001.zip › Combined Data/Calibration/Calibration thermal/rgb83.png]

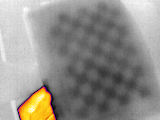

Supplement: Supplementary file 1 [file sensors-23-00560-s001.zip › Combined Data/Calibration/Calibration thermal/rgb84.png]

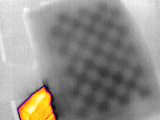

Supplement: Supplementary file 1 [file sensors-23-00560-s001.zip › Combined Data/Calibration/Calibration thermal/rgb85.png]

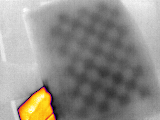

Supplement: Supplementary file 1 [file sensors-23-00560-s001.zip › Combined Data/Calibration/Calibration thermal/rgb86.png]

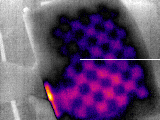

Supplement: Supplementary file 1 [file sensors-23-00560-s001.zip › Combined Data/Calibration/Calibration thermal/rgb87.png]

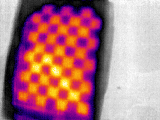

Supplement: Supplementary file 1 [file sensors-23-00560-s001.zip › Combined Data/Calibration/Calibration thermal/rgb90.png]

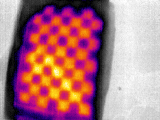

Supplement: Supplementary file 1 [file sensors-23-00560-s001.zip › Combined Data/Calibration/Calibration thermal/rgb91.png]

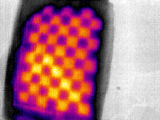

Supplement: Supplementary file 1 [file sensors-23-00560-s001.zip › Combined Data/Calibration/Calibration thermal/rgb92.png]
