# Supplementary figures and images for: Food Image Segmentation Using Multi-Modal Imaging Sensors with Color and Thermal Data (part 1 of 2)
Source: Sensors (Basel). 2023 Jan 4;23(2):560. doi: 10.3390/s23020560 (PMC9860575; doi:10.3390/s23020560)

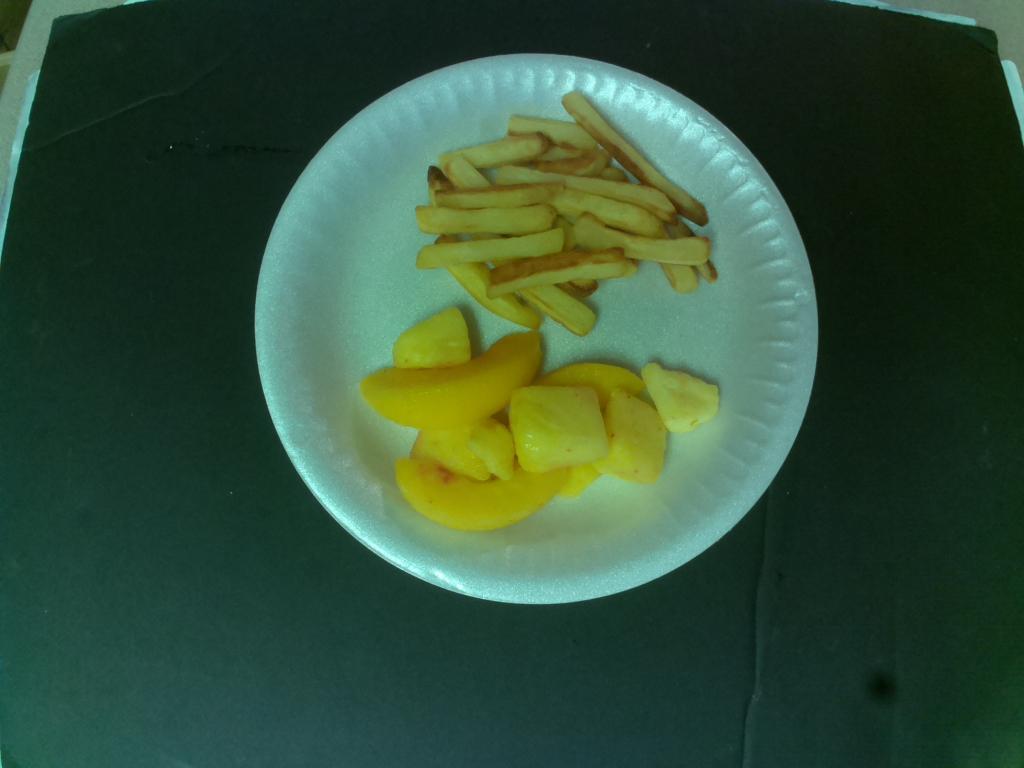

Supplement: Supplementary file 1 [file sensors-23-00560-s001.zip › Combined Data/Additional RGB+THERMAL/1.jpeg]

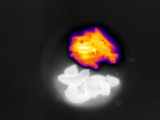

Supplement: Supplementary file 1 [file sensors-23-00560-s001.zip › Combined Data/Additional RGB+THERMAL/1.png]

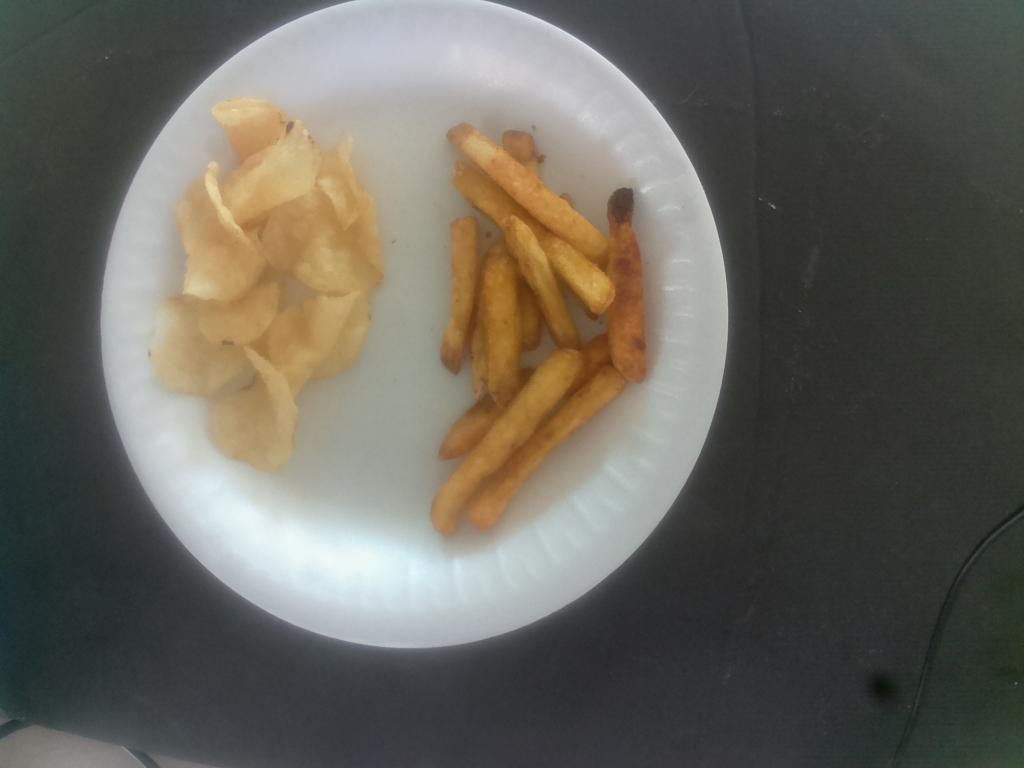

Supplement: Supplementary file 1 [file sensors-23-00560-s001.zip › Combined Data/C1 temporal/0/0.jpeg]

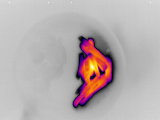

Supplement: Supplementary file 1 [file sensors-23-00560-s001.zip › Combined Data/C1 temporal/0/0.png]

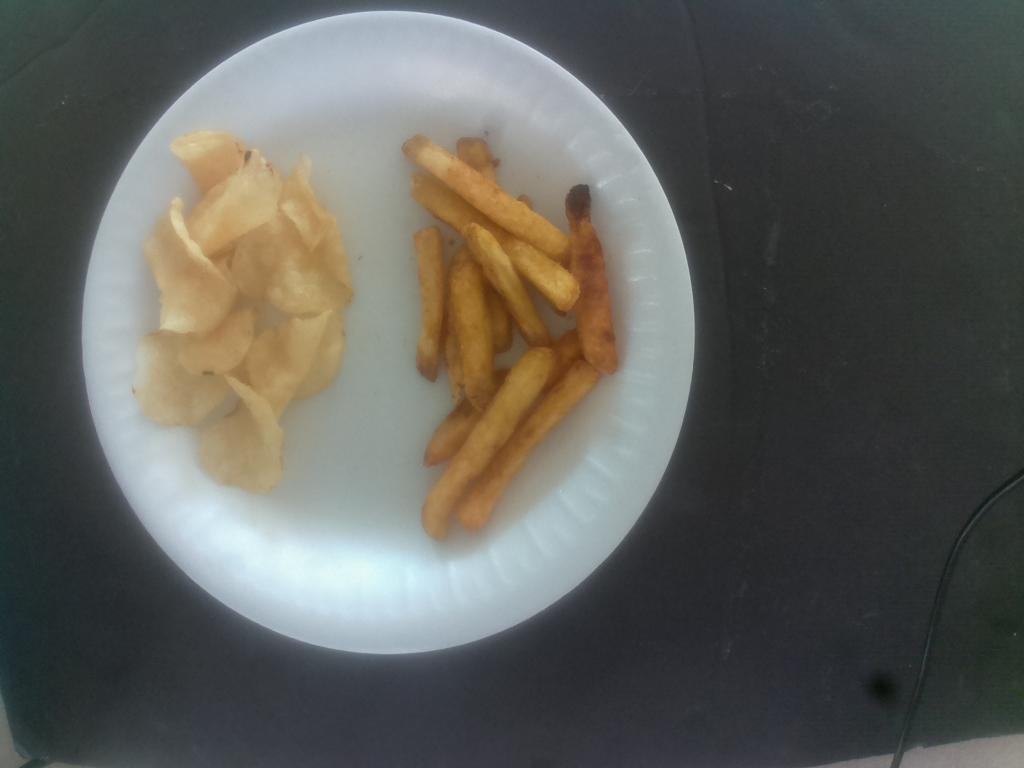

Supplement: Supplementary file 1 [file sensors-23-00560-s001.zip › Combined Data/C1 temporal/105sec/7.jpeg]

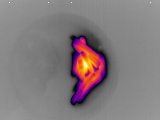

Supplement: Supplementary file 1 [file sensors-23-00560-s001.zip › Combined Data/C1 temporal/105sec/7.png]

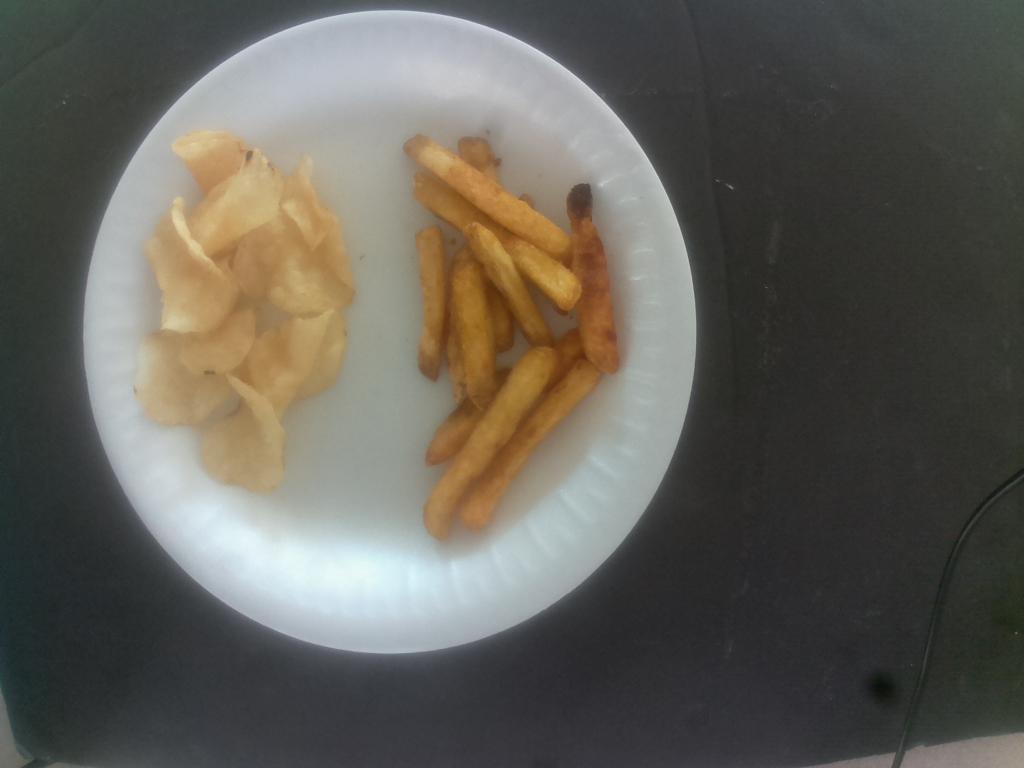

Supplement: Supplementary file 1 [file sensors-23-00560-s001.zip › Combined Data/C1 temporal/120sec/8.jpeg]

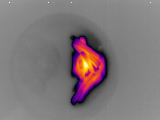

Supplement: Supplementary file 1 [file sensors-23-00560-s001.zip › Combined Data/C1 temporal/120sec/8.png]

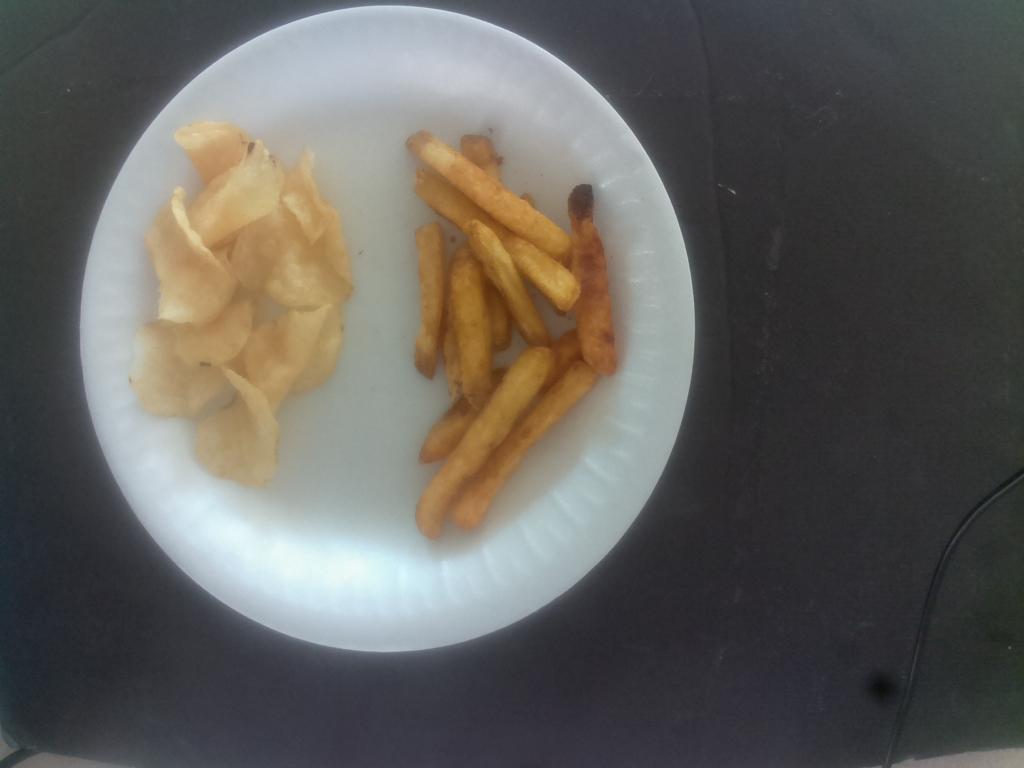

Supplement: Supplementary file 1 [file sensors-23-00560-s001.zip › Combined Data/C1 temporal/135sec/9.jpeg]

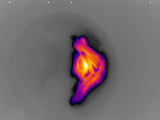

Supplement: Supplementary file 1 [file sensors-23-00560-s001.zip › Combined Data/C1 temporal/135sec/9.png]

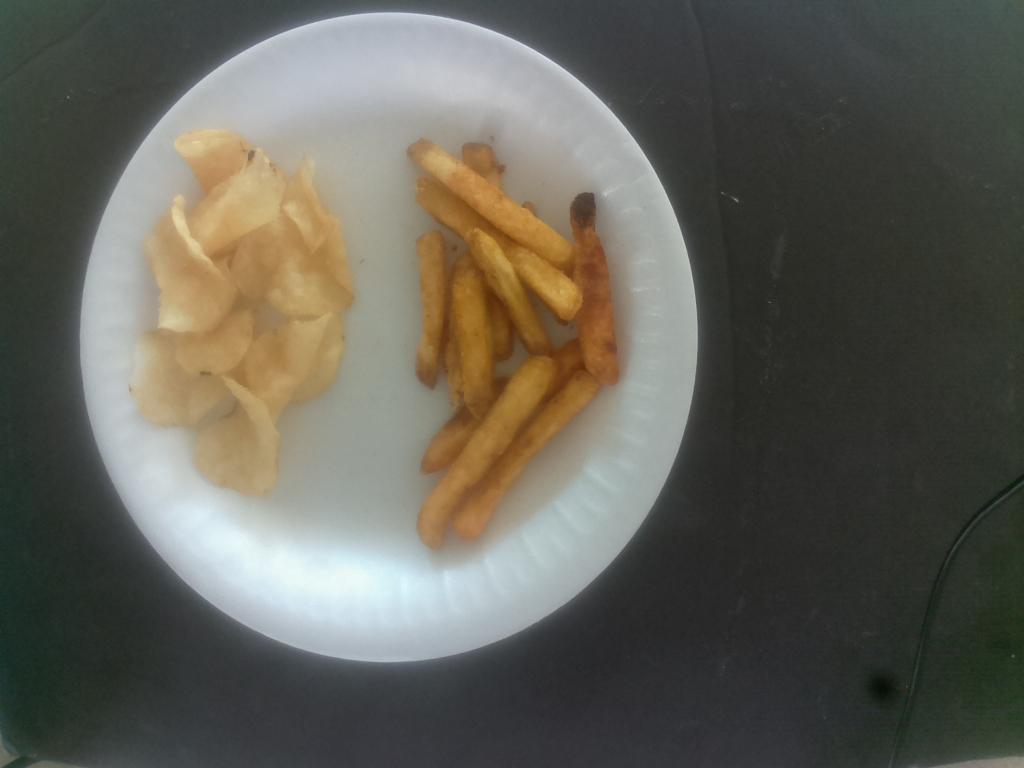

Supplement: Supplementary file 1 [file sensors-23-00560-s001.zip › Combined Data/C1 temporal/150sec/10.jpeg]

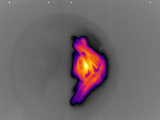

Supplement: Supplementary file 1 [file sensors-23-00560-s001.zip › Combined Data/C1 temporal/150sec/10.png]

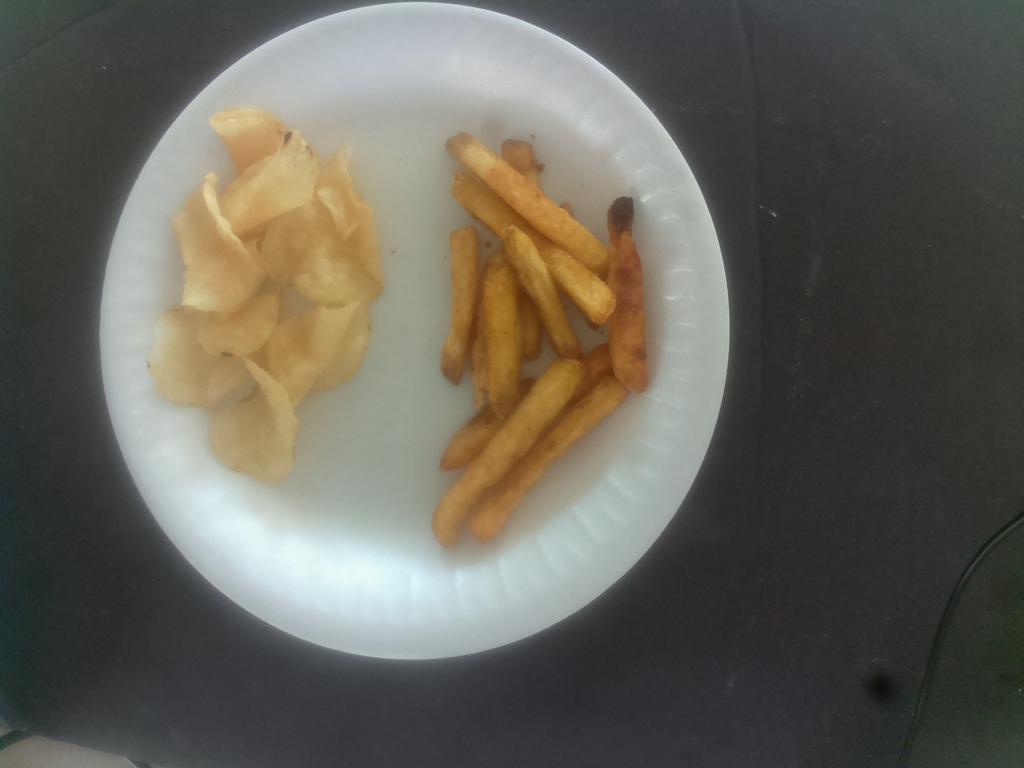

Supplement: Supplementary file 1 [file sensors-23-00560-s001.zip › Combined Data/C1 temporal/15sec/1.jpeg]

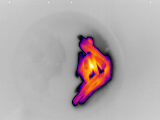

Supplement: Supplementary file 1 [file sensors-23-00560-s001.zip › Combined Data/C1 temporal/15sec/1.png]

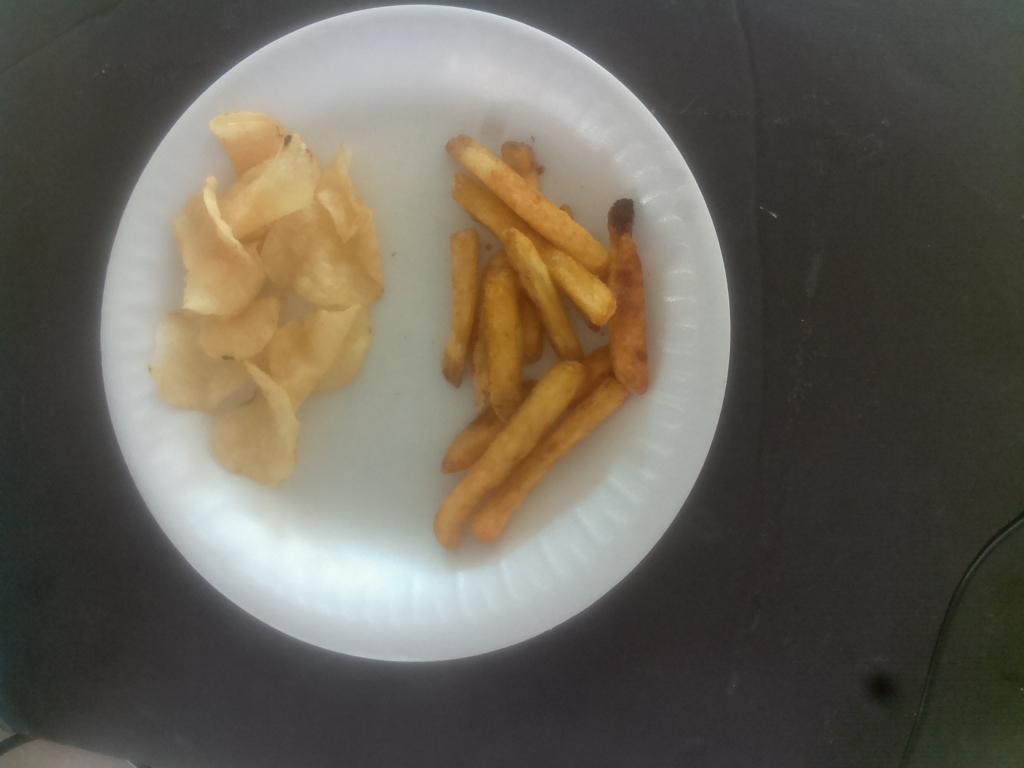

Supplement: Supplementary file 1 [file sensors-23-00560-s001.zip › Combined Data/C1 temporal/30sec/2.jpeg]

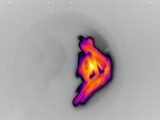

Supplement: Supplementary file 1 [file sensors-23-00560-s001.zip › Combined Data/C1 temporal/30sec/2.png]

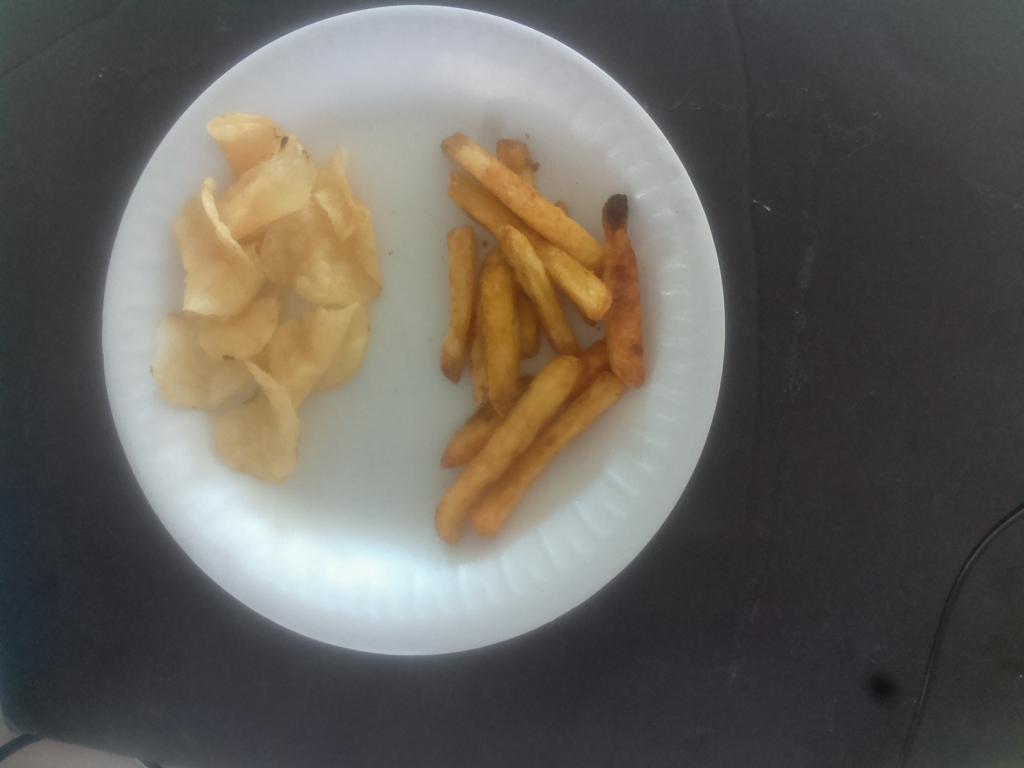

Supplement: Supplementary file 1 [file sensors-23-00560-s001.zip › Combined Data/C1 temporal/45sec/3.jpeg]

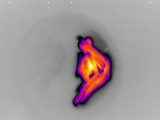

Supplement: Supplementary file 1 [file sensors-23-00560-s001.zip › Combined Data/C1 temporal/45sec/3.png]

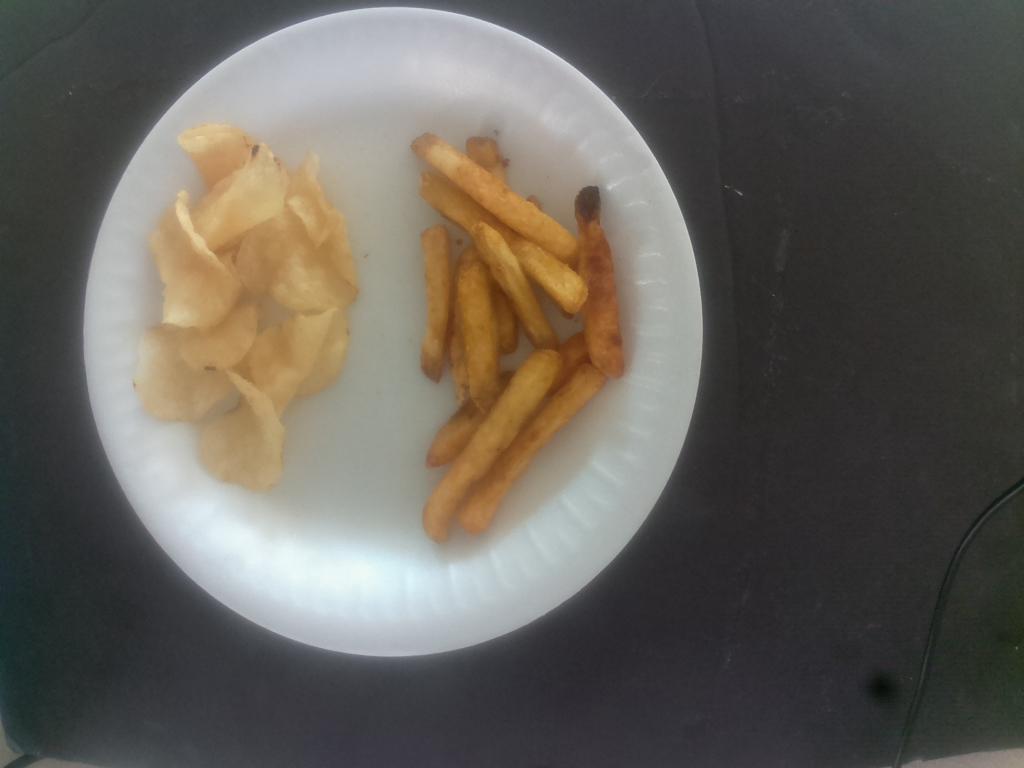

Supplement: Supplementary file 1 [file sensors-23-00560-s001.zip › Combined Data/C1 temporal/60sec/4.jpeg]

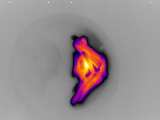

Supplement: Supplementary file 1 [file sensors-23-00560-s001.zip › Combined Data/C1 temporal/60sec/4.png]

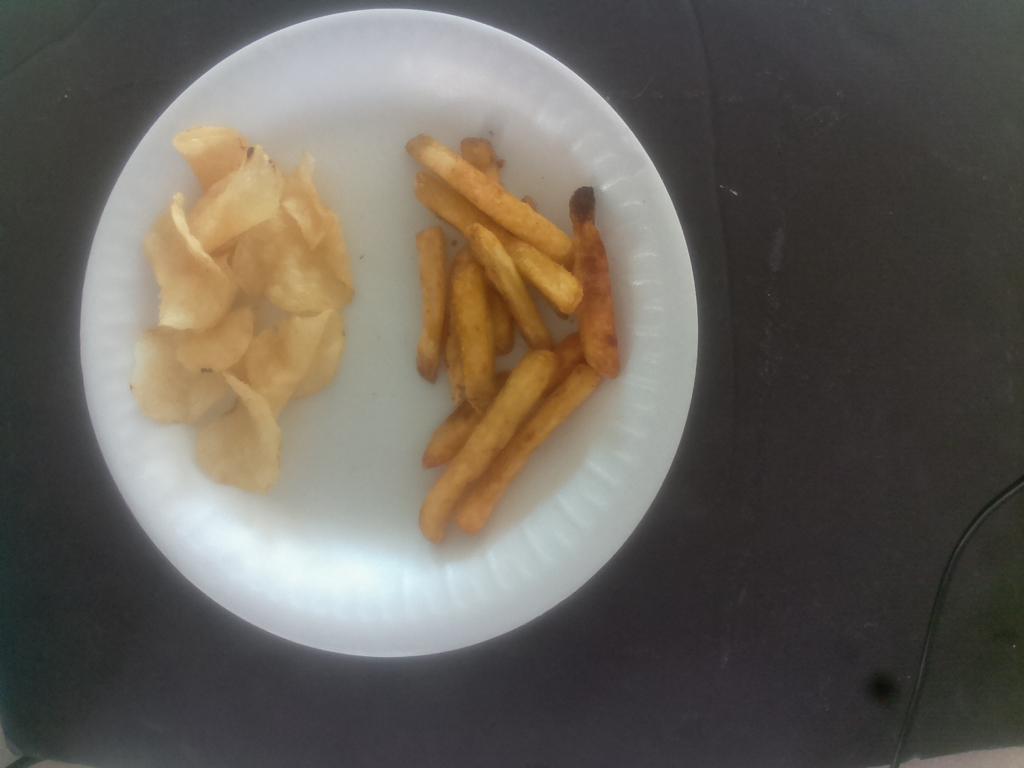

Supplement: Supplementary file 1 [file sensors-23-00560-s001.zip › Combined Data/C1 temporal/75sec/5.jpeg]

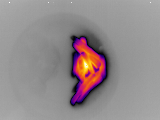

Supplement: Supplementary file 1 [file sensors-23-00560-s001.zip › Combined Data/C1 temporal/75sec/5.png]

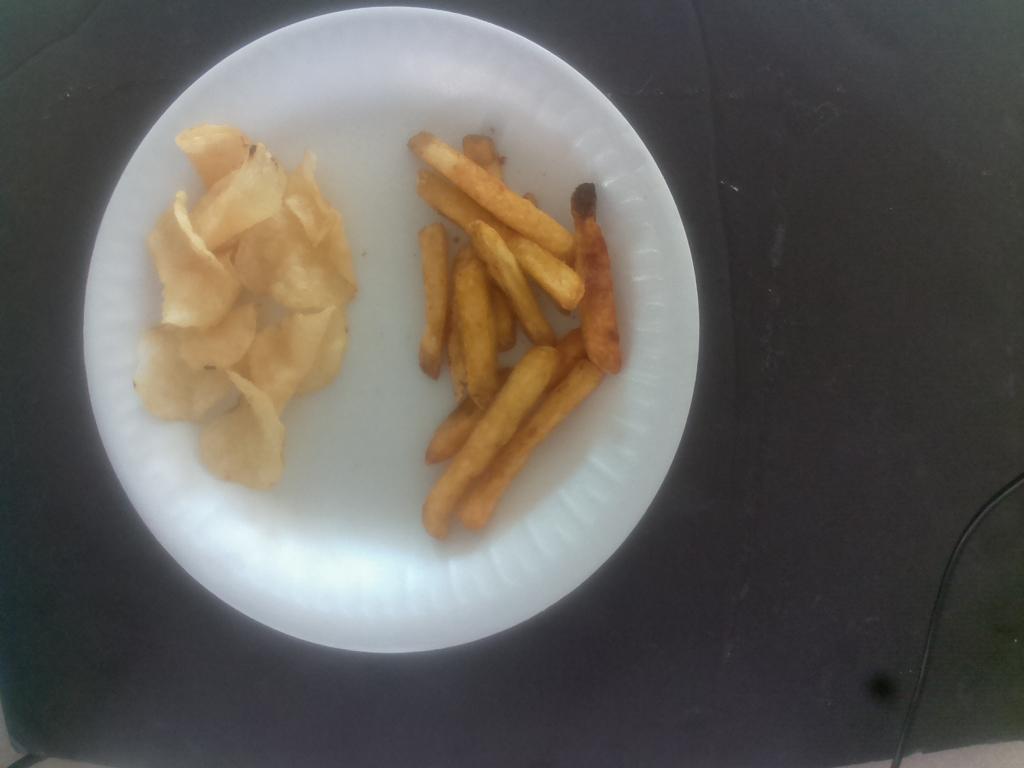

Supplement: Supplementary file 1 [file sensors-23-00560-s001.zip › Combined Data/C1 temporal/90sec/6.jpeg]

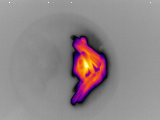

Supplement: Supplementary file 1 [file sensors-23-00560-s001.zip › Combined Data/C1 temporal/90sec/6.png]

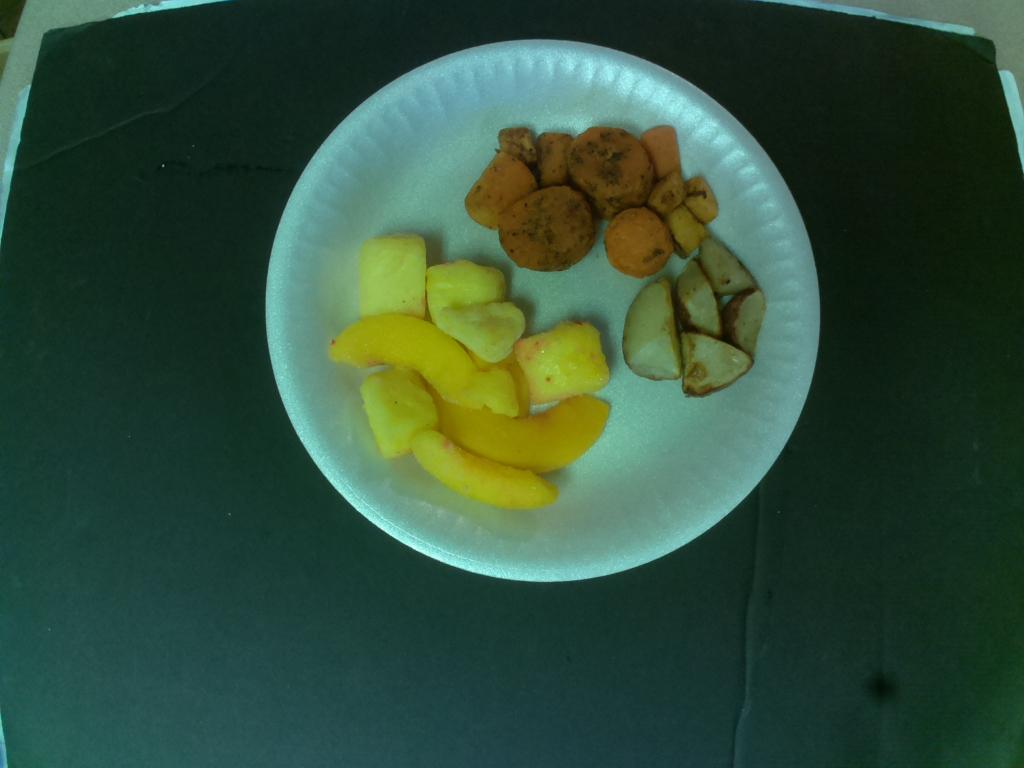

Supplement: Supplementary file 1 [file sensors-23-00560-s001.zip › Combined Data/C2 RGB+THERMAL/0.jpeg]

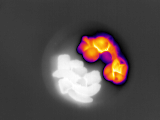

Supplement: Supplementary file 1 [file sensors-23-00560-s001.zip › Combined Data/C2 RGB+THERMAL/0.png]

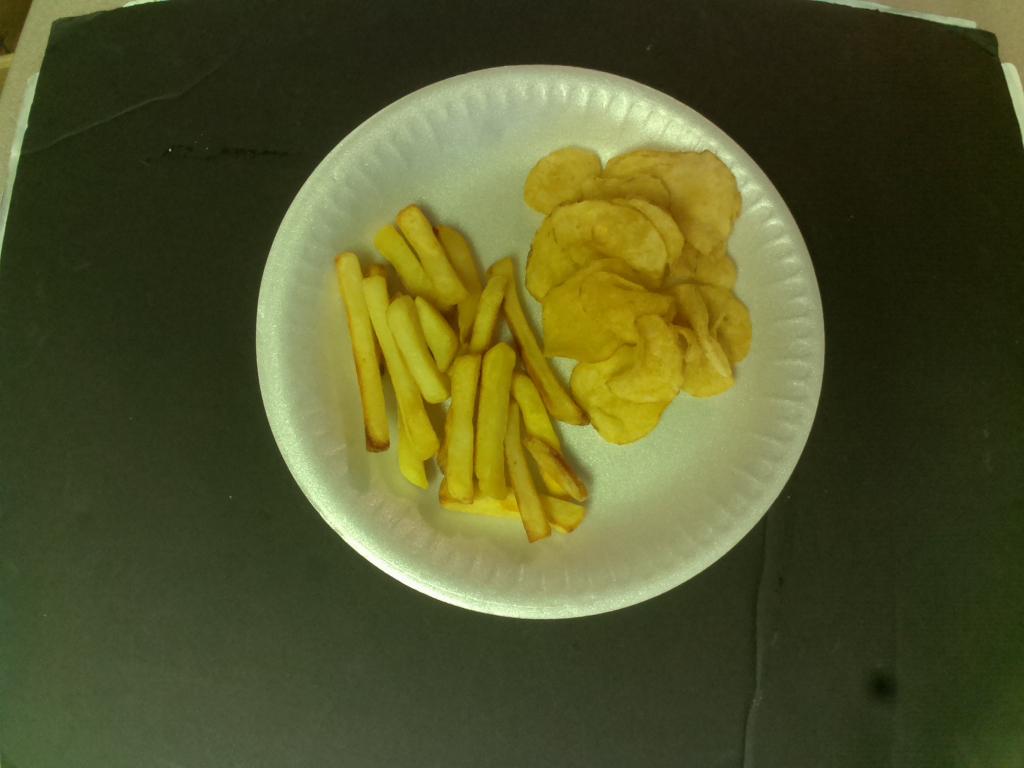

Supplement: Supplementary file 1 [file sensors-23-00560-s001.zip › Combined Data/C3 RGB+THERMAL/2.jpeg]

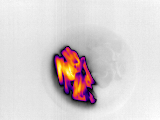

Supplement: Supplementary file 1 [file sensors-23-00560-s001.zip › Combined Data/C3 RGB+THERMAL/2.png]

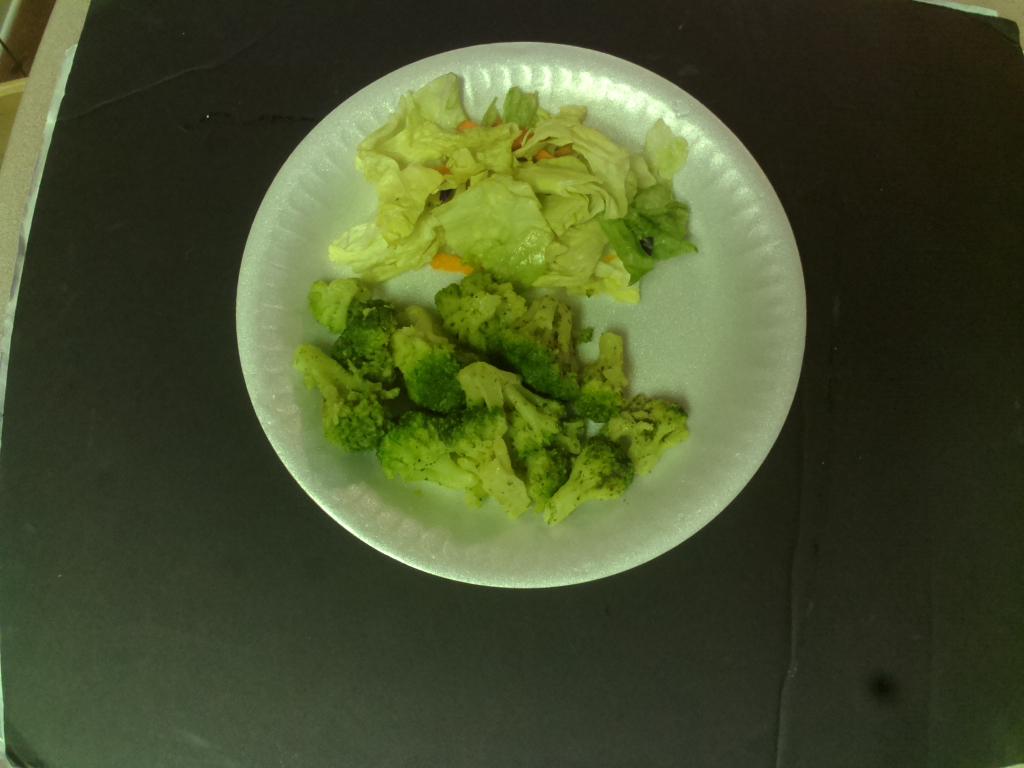

Supplement: Supplementary file 1 [file sensors-23-00560-s001.zip › Combined Data/C4 RGB+THERMAL/3.jpeg]

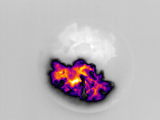

Supplement: Supplementary file 1 [file sensors-23-00560-s001.zip › Combined Data/C4 RGB+THERMAL/3.png]

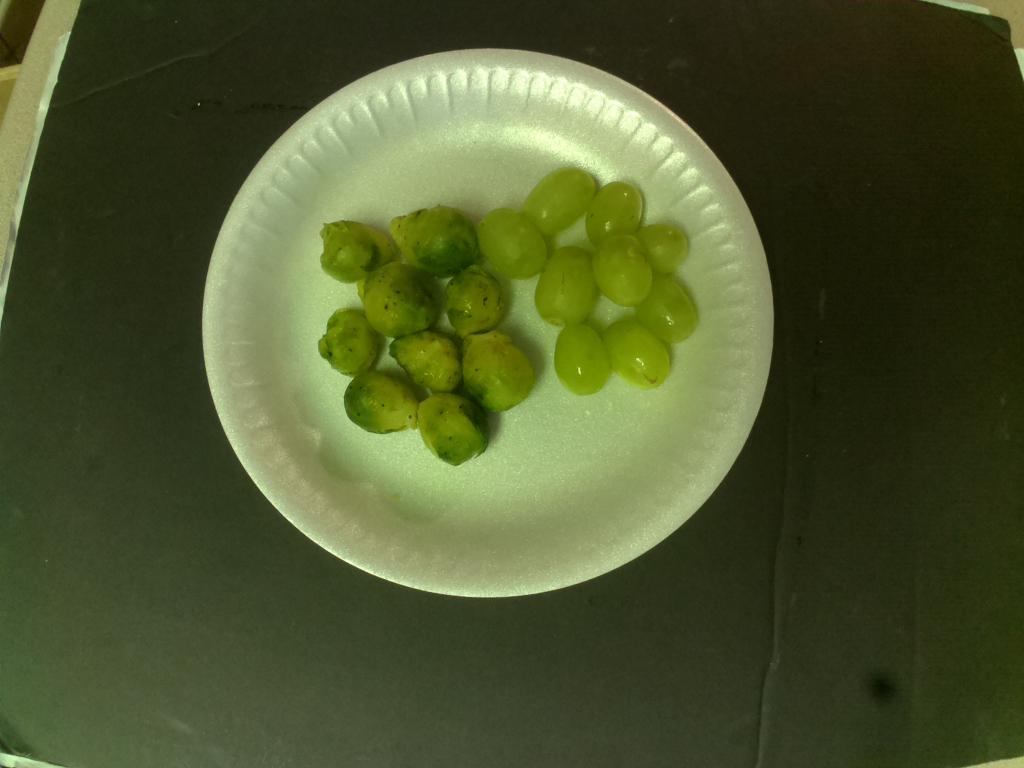

Supplement: Supplementary file 1 [file sensors-23-00560-s001.zip › Combined Data/C5 RGB+THERMAL/4.jpeg]

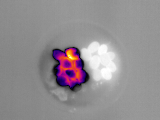

Supplement: Supplementary file 1 [file sensors-23-00560-s001.zip › Combined Data/C5 RGB+THERMAL/4.png]

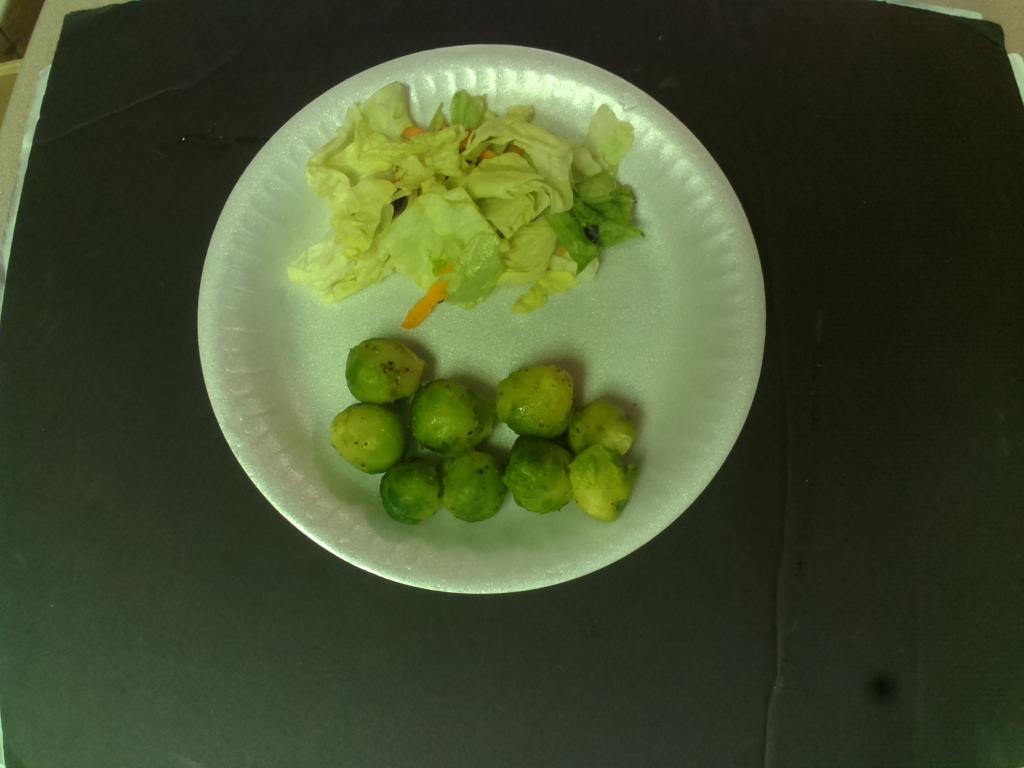

Supplement: Supplementary file 1 [file sensors-23-00560-s001.zip › Combined Data/C6 RGB+THERMAL/5.jpeg]

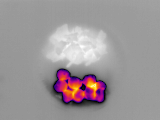

Supplement: Supplementary file 1 [file sensors-23-00560-s001.zip › Combined Data/C6 RGB+THERMAL/5.png]

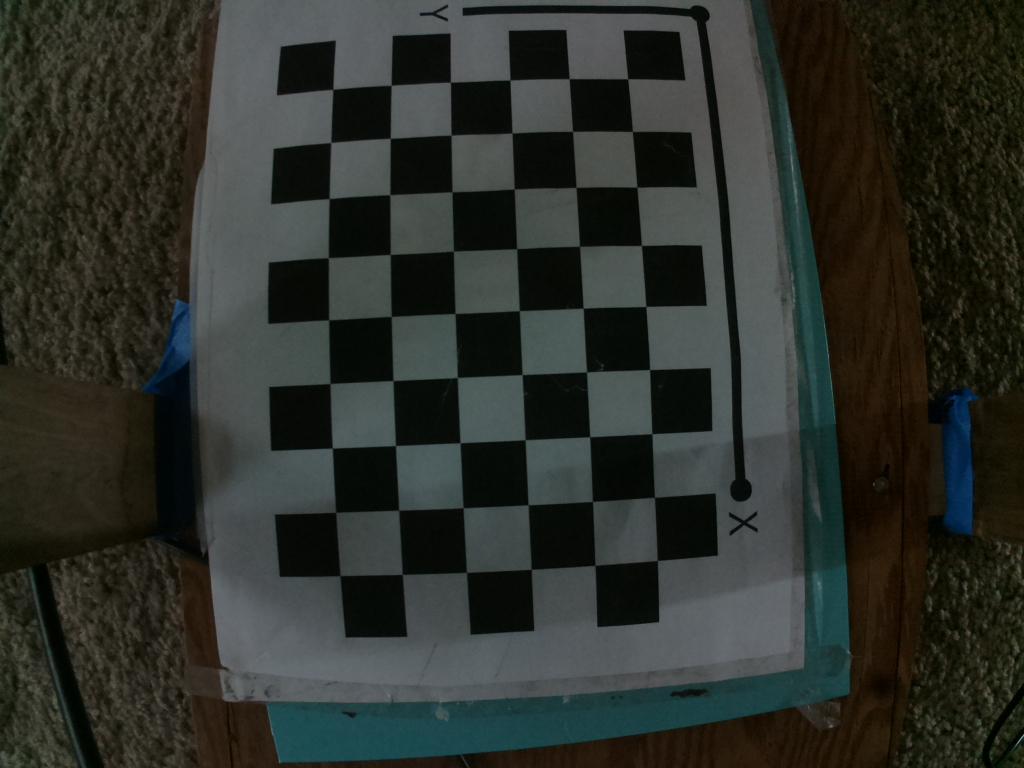

Supplement: Supplementary file 1 [file sensors-23-00560-s001.zip › Combined Data/Calibration/Calibration picam/0 (2).jpeg]

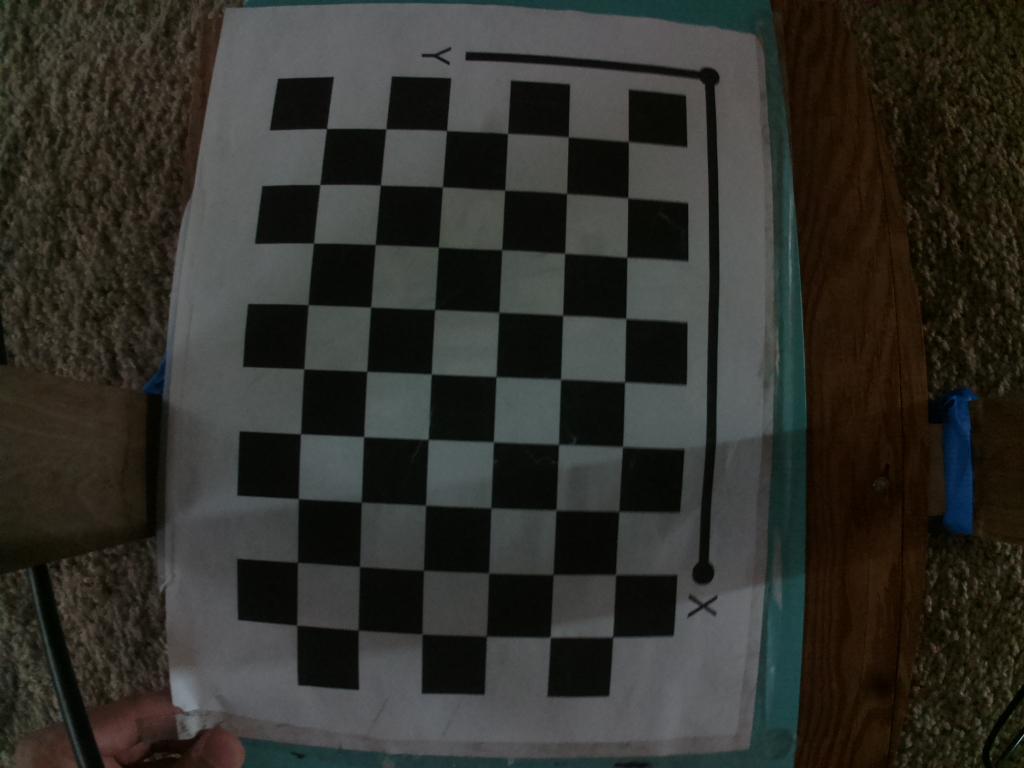

Supplement: Supplementary file 1 [file sensors-23-00560-s001.zip › Combined Data/Calibration/Calibration picam/0 (3).jpeg]

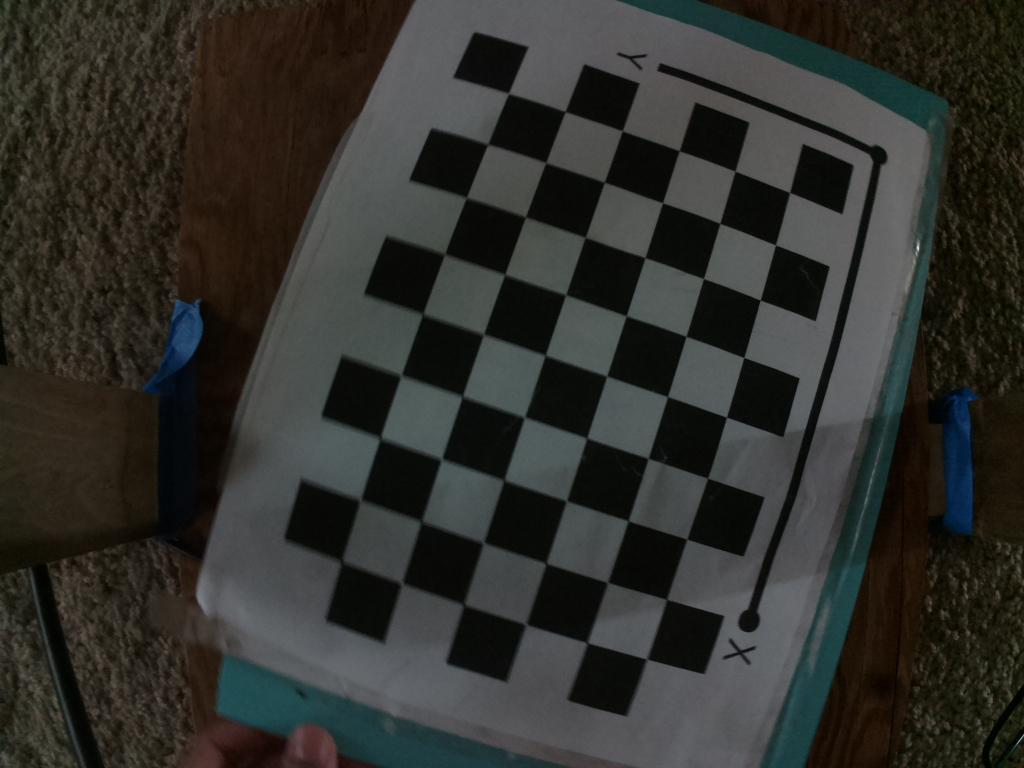

Supplement: Supplementary file 1 [file sensors-23-00560-s001.zip › Combined Data/Calibration/Calibration picam/0 (4).jpeg]

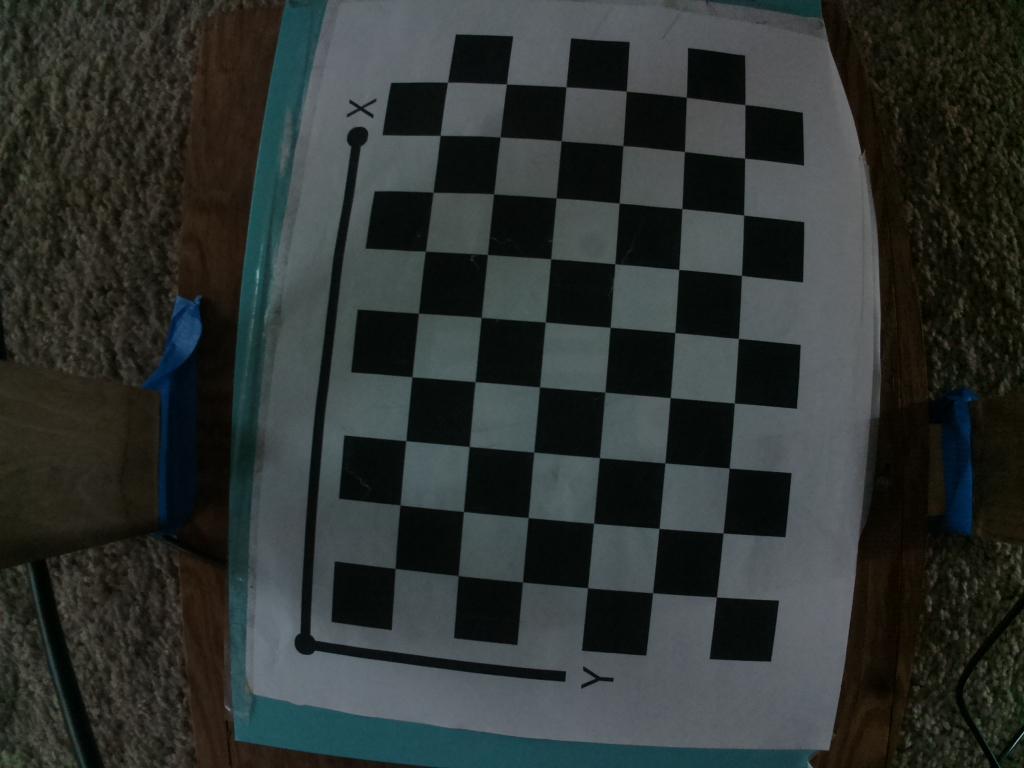

Supplement: Supplementary file 1 [file sensors-23-00560-s001.zip › Combined Data/Calibration/Calibration picam/0 (5).jpeg]

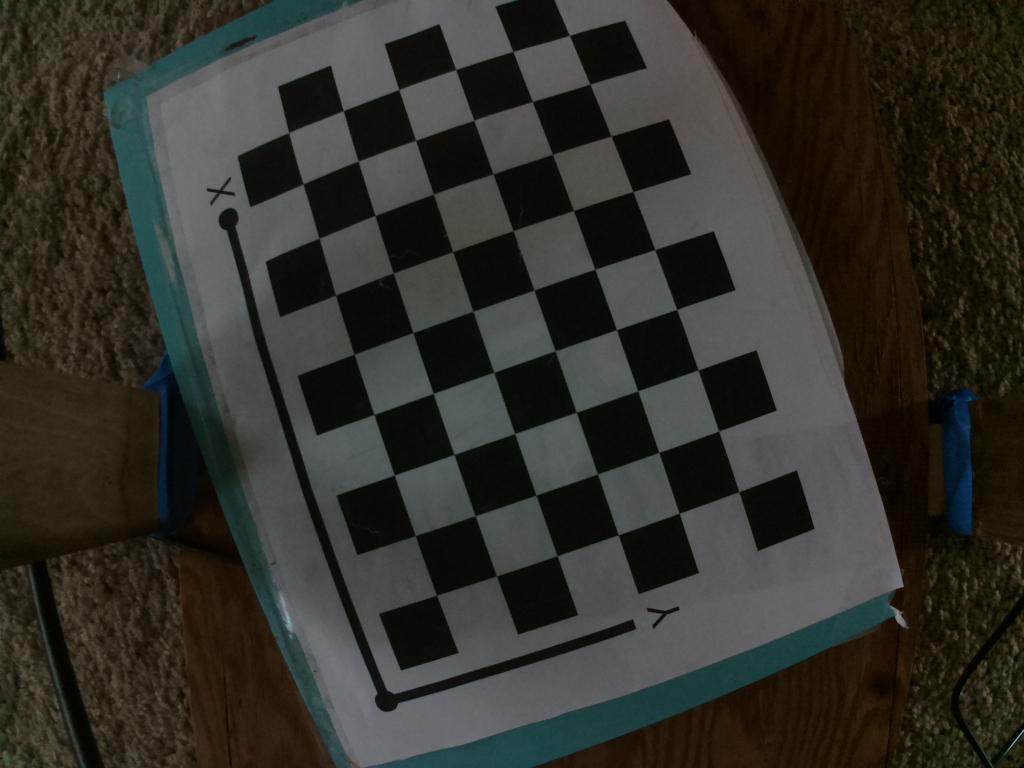

Supplement: Supplementary file 1 [file sensors-23-00560-s001.zip › Combined Data/Calibration/Calibration picam/0 (6).jpeg]

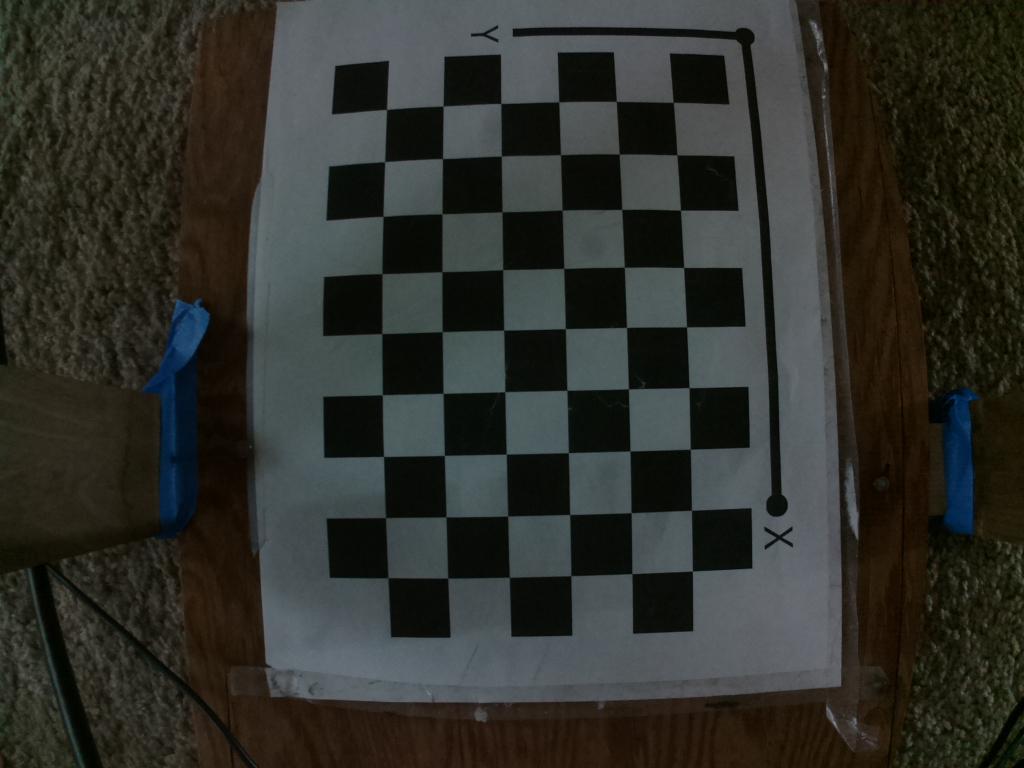

Supplement: Supplementary file 1 [file sensors-23-00560-s001.zip › Combined Data/Calibration/Calibration picam/0.jpeg]

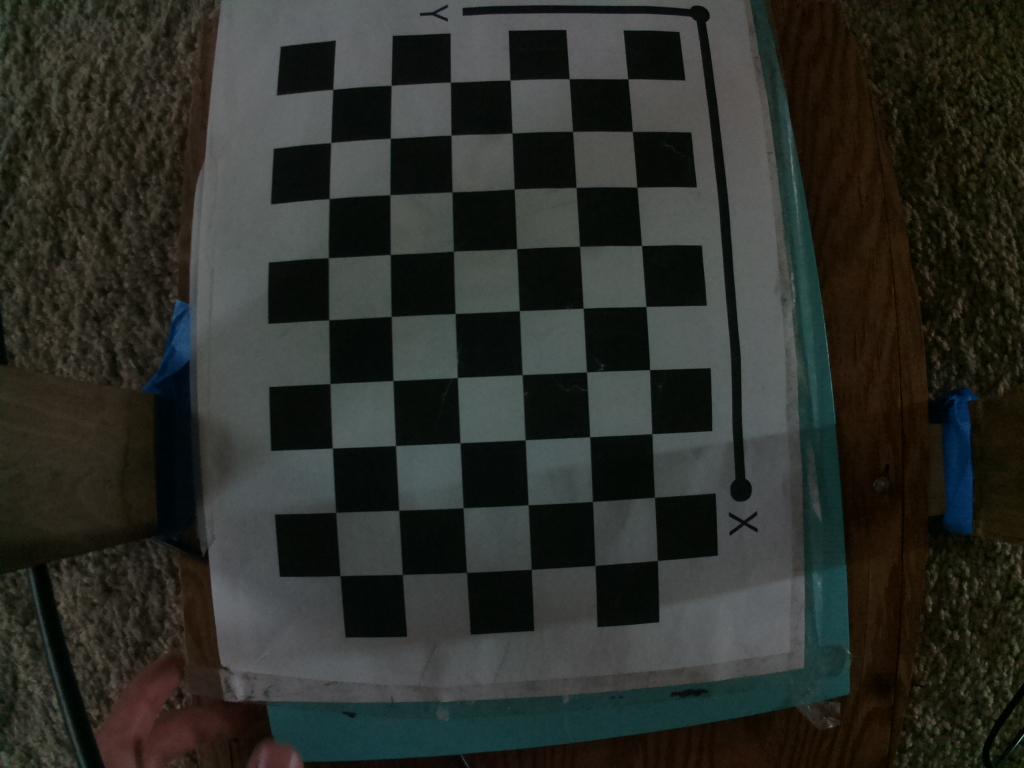

Supplement: Supplementary file 1 [file sensors-23-00560-s001.zip › Combined Data/Calibration/Calibration picam/1 (2).jpeg]

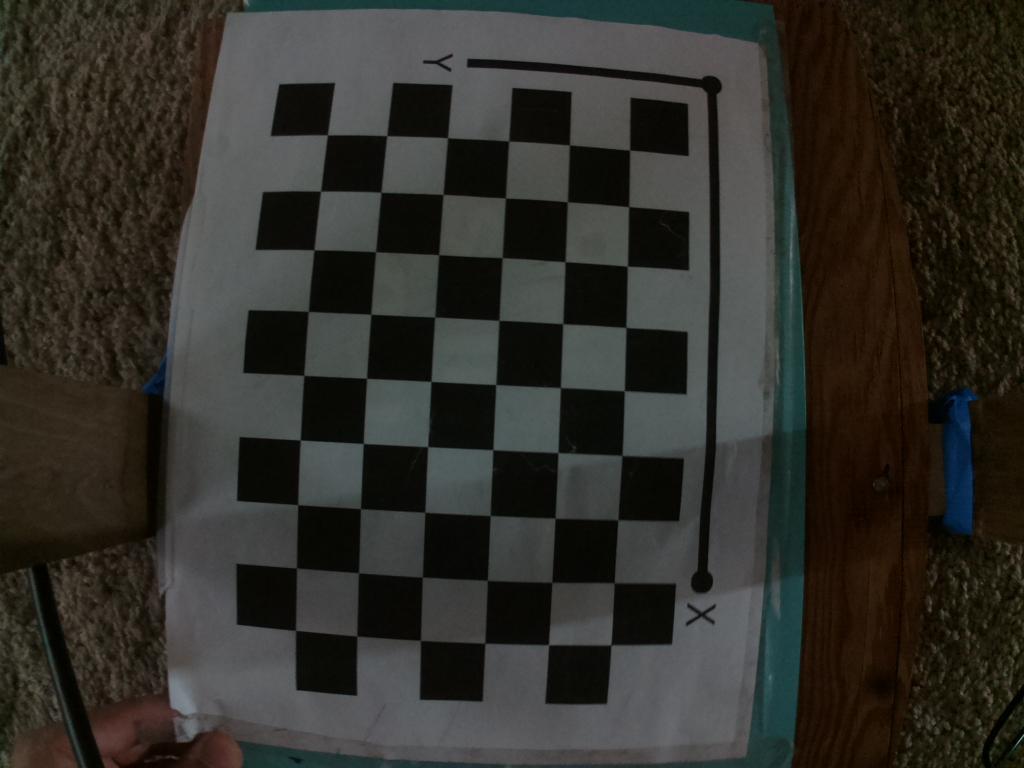

Supplement: Supplementary file 1 [file sensors-23-00560-s001.zip › Combined Data/Calibration/Calibration picam/1 (3).jpeg]

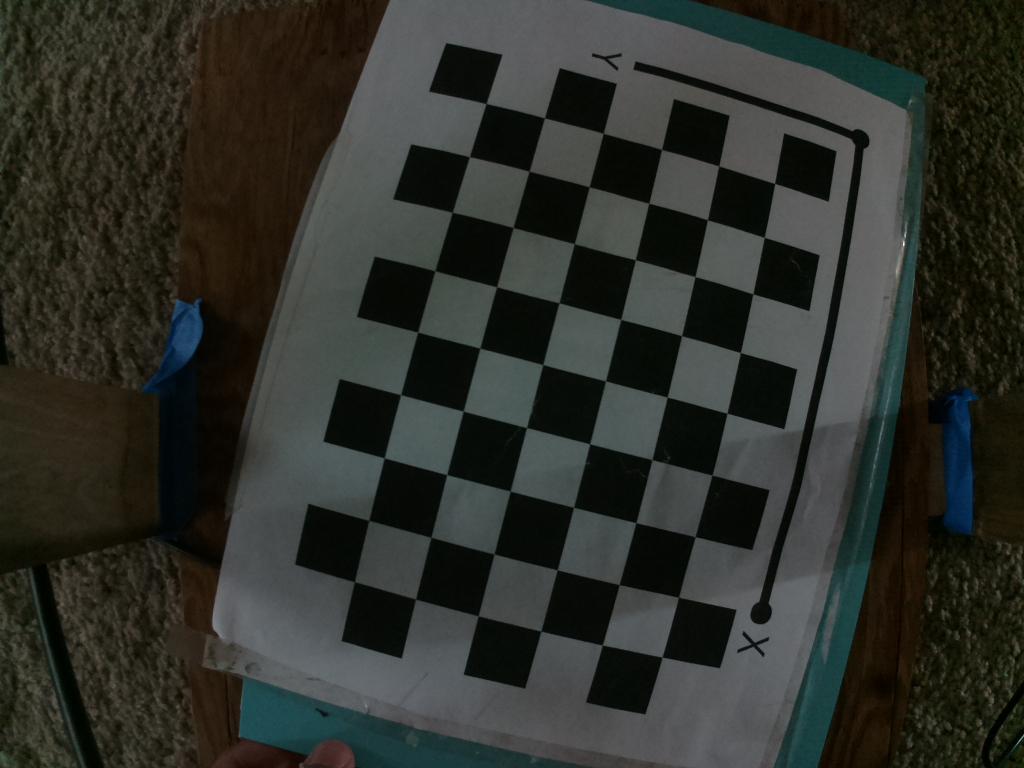

Supplement: Supplementary file 1 [file sensors-23-00560-s001.zip › Combined Data/Calibration/Calibration picam/1 (4).jpeg]

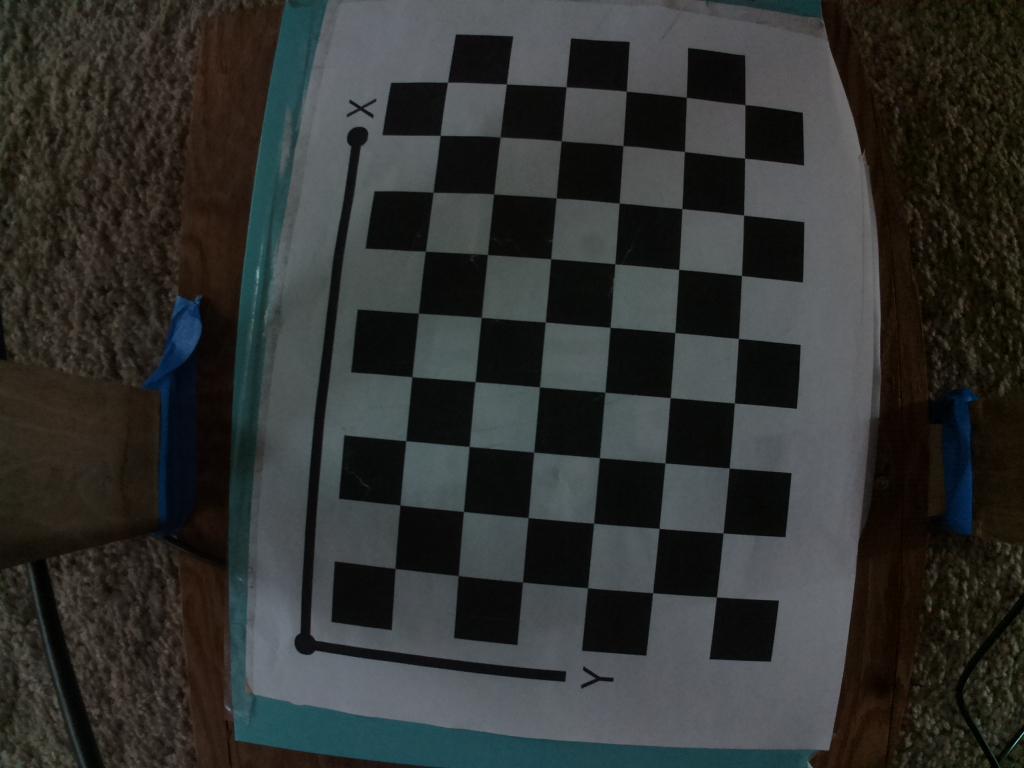

Supplement: Supplementary file 1 [file sensors-23-00560-s001.zip › Combined Data/Calibration/Calibration picam/1 (5).jpeg]

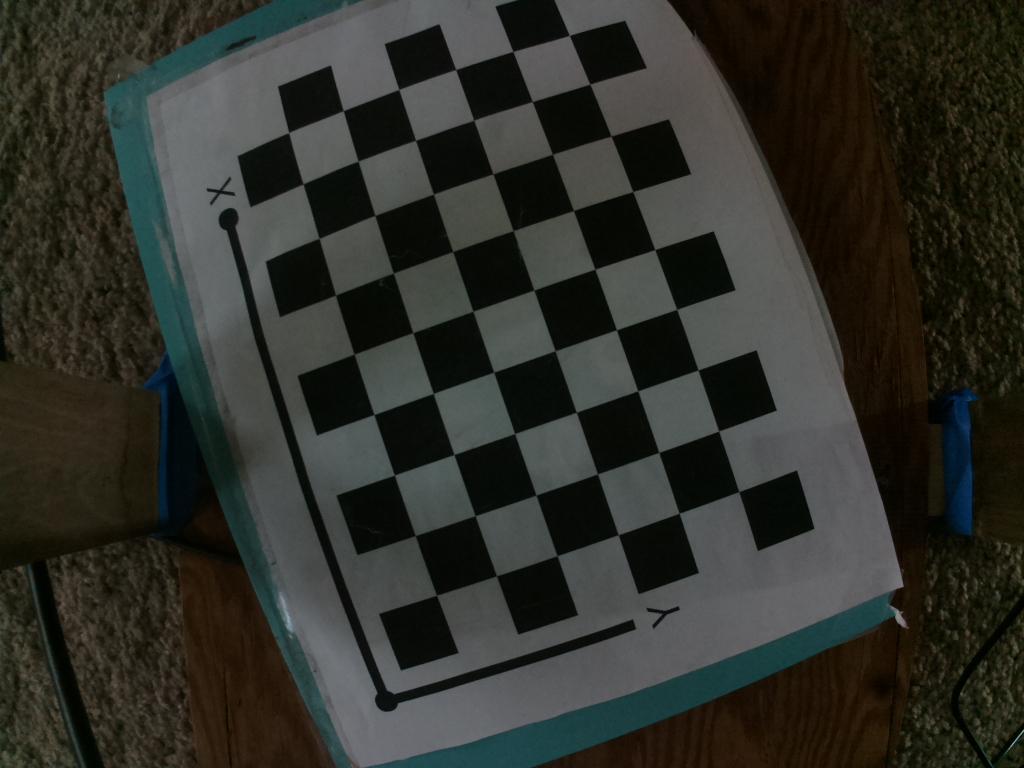

Supplement: Supplementary file 1 [file sensors-23-00560-s001.zip › Combined Data/Calibration/Calibration picam/1 (6).jpeg]

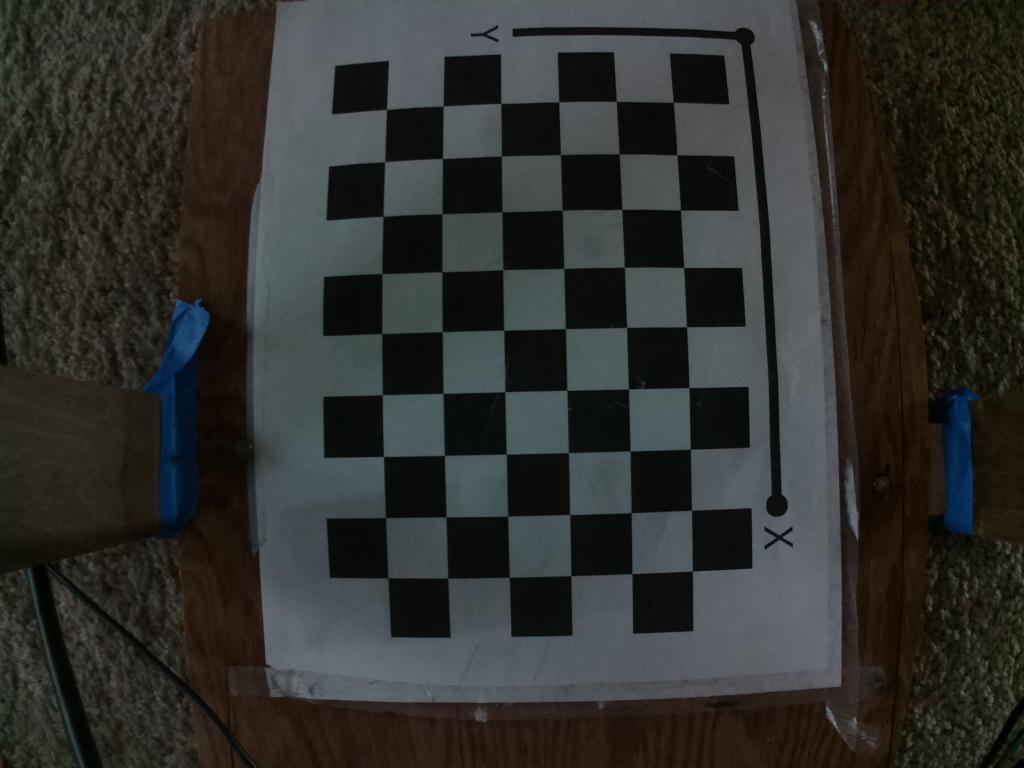

Supplement: Supplementary file 1 [file sensors-23-00560-s001.zip › Combined Data/Calibration/Calibration picam/1.jpeg]

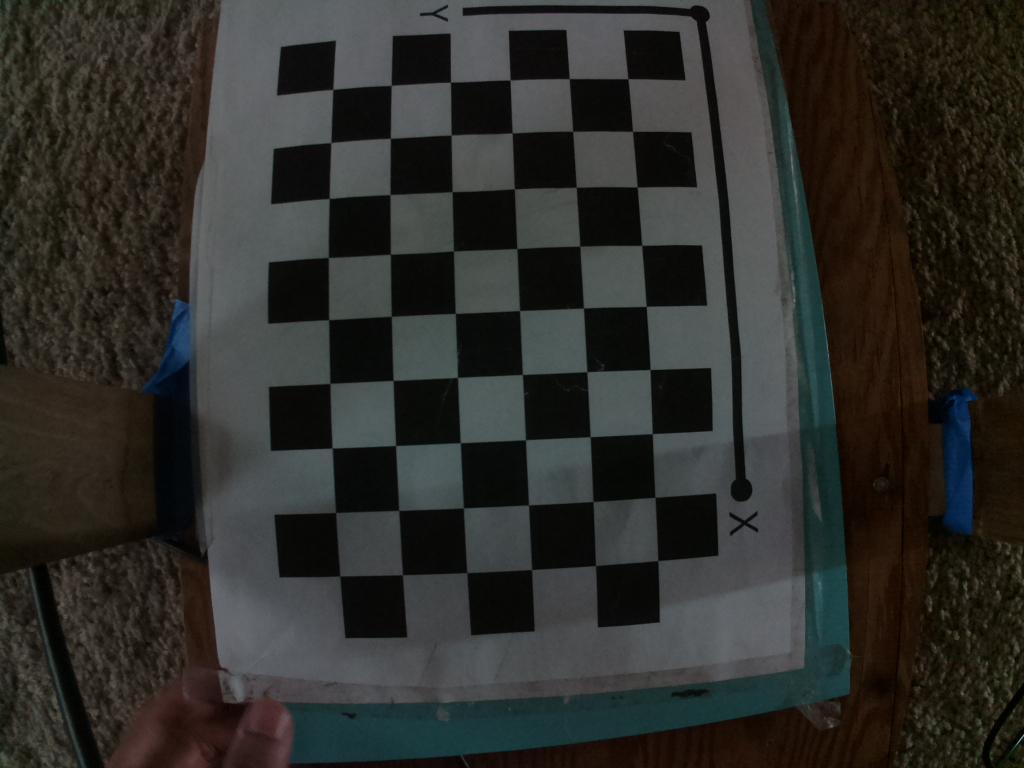

Supplement: Supplementary file 1 [file sensors-23-00560-s001.zip › Combined Data/Calibration/Calibration picam/2 (2).jpeg]

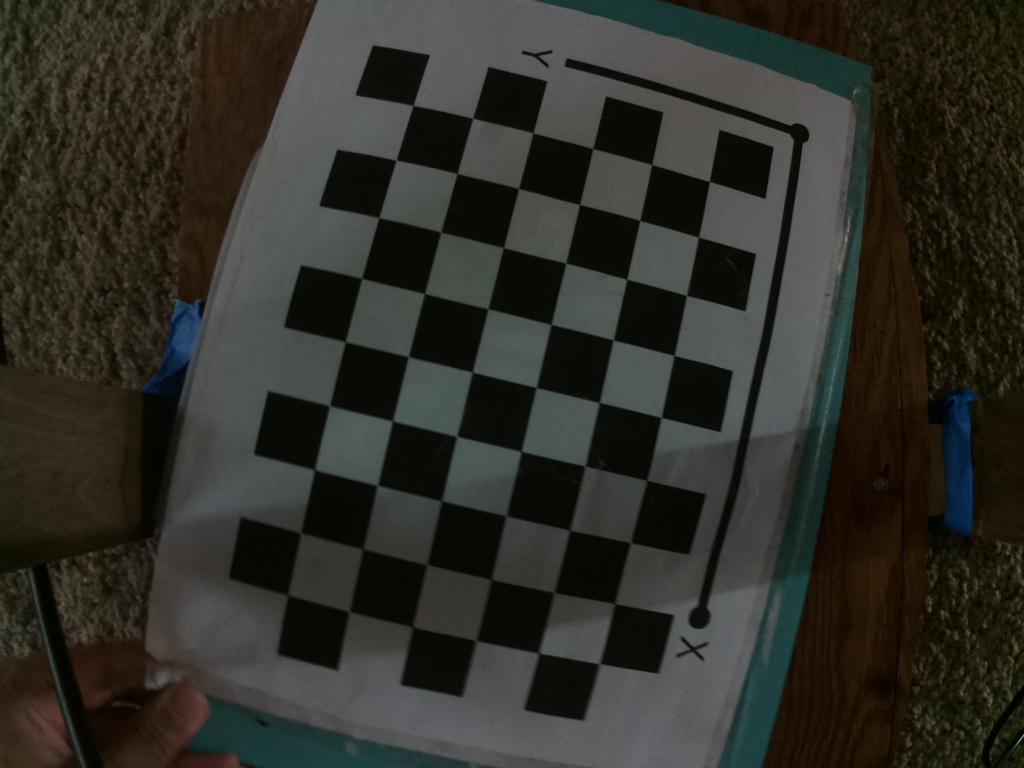

Supplement: Supplementary file 1 [file sensors-23-00560-s001.zip › Combined Data/Calibration/Calibration picam/2 (3).jpeg]

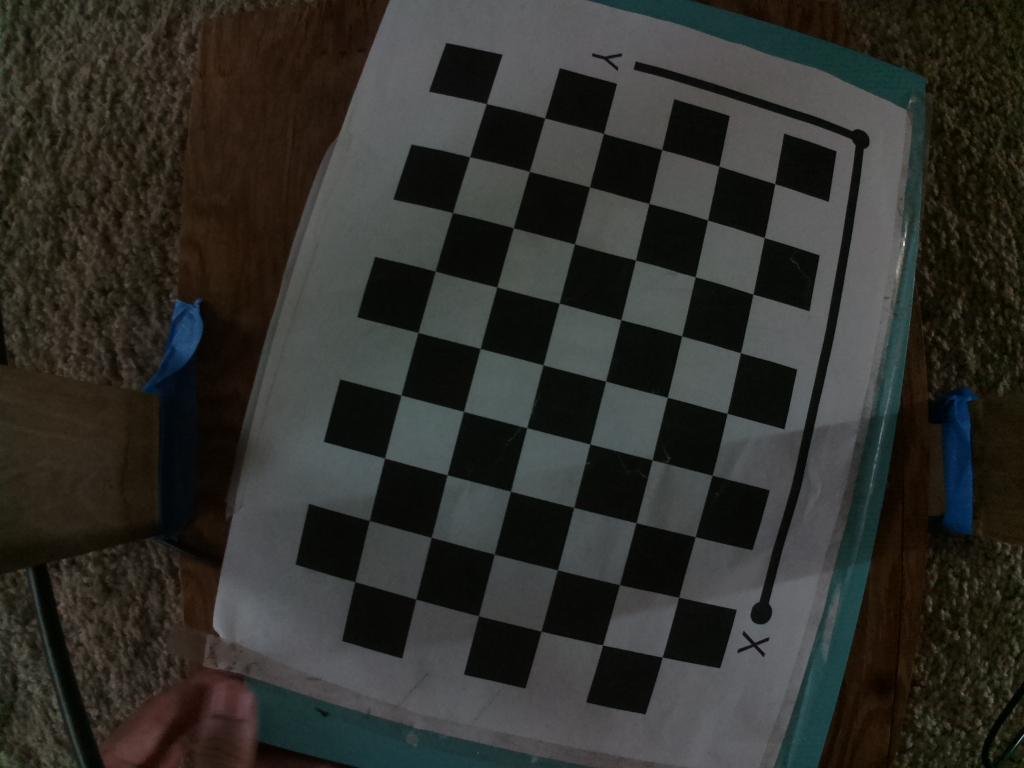

Supplement: Supplementary file 1 [file sensors-23-00560-s001.zip › Combined Data/Calibration/Calibration picam/2 (4).jpeg]

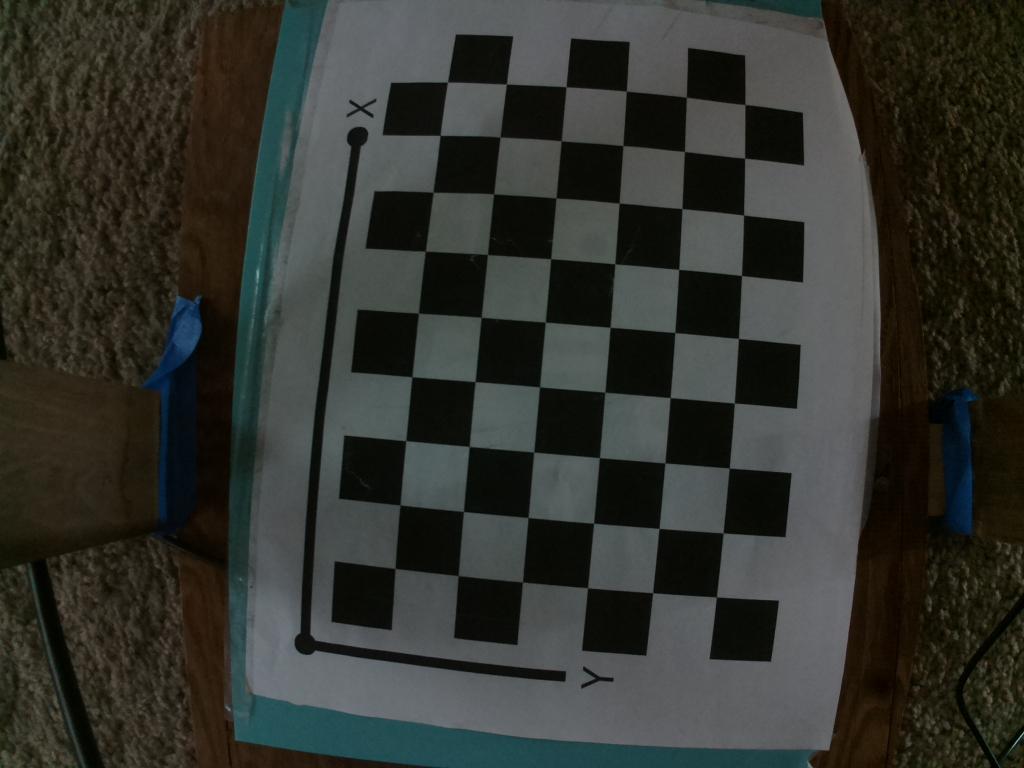

Supplement: Supplementary file 1 [file sensors-23-00560-s001.zip › Combined Data/Calibration/Calibration picam/2 (5).jpeg]

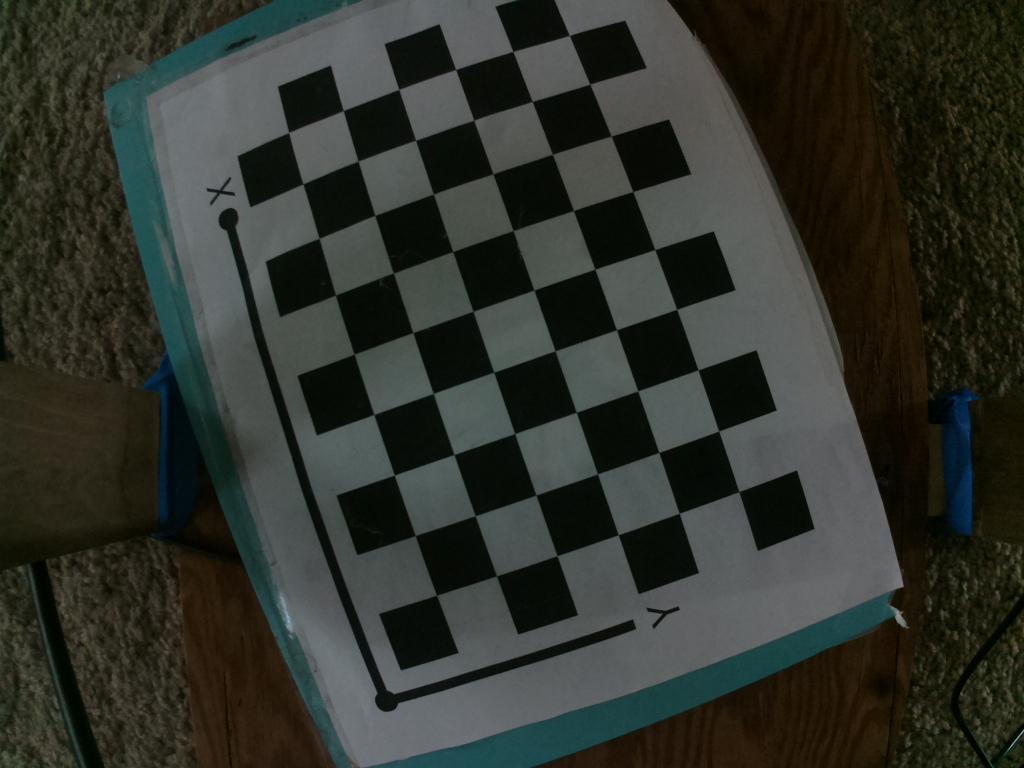

Supplement: Supplementary file 1 [file sensors-23-00560-s001.zip › Combined Data/Calibration/Calibration picam/2 (6).jpeg]

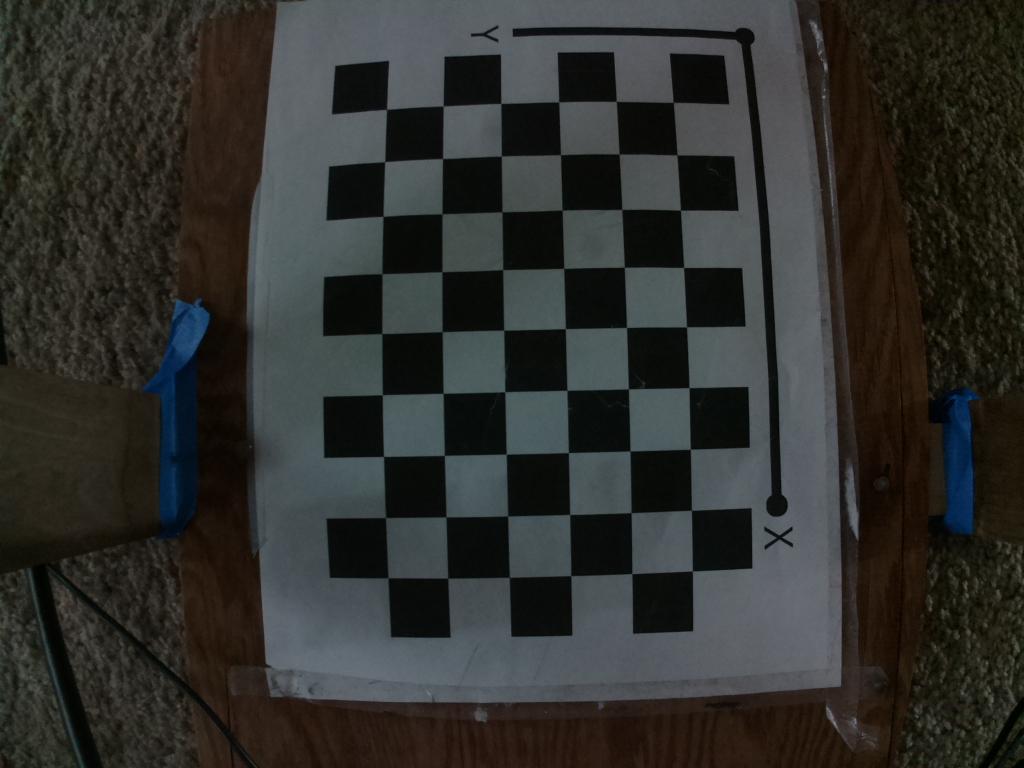

Supplement: Supplementary file 1 [file sensors-23-00560-s001.zip › Combined Data/Calibration/Calibration picam/2.jpeg]

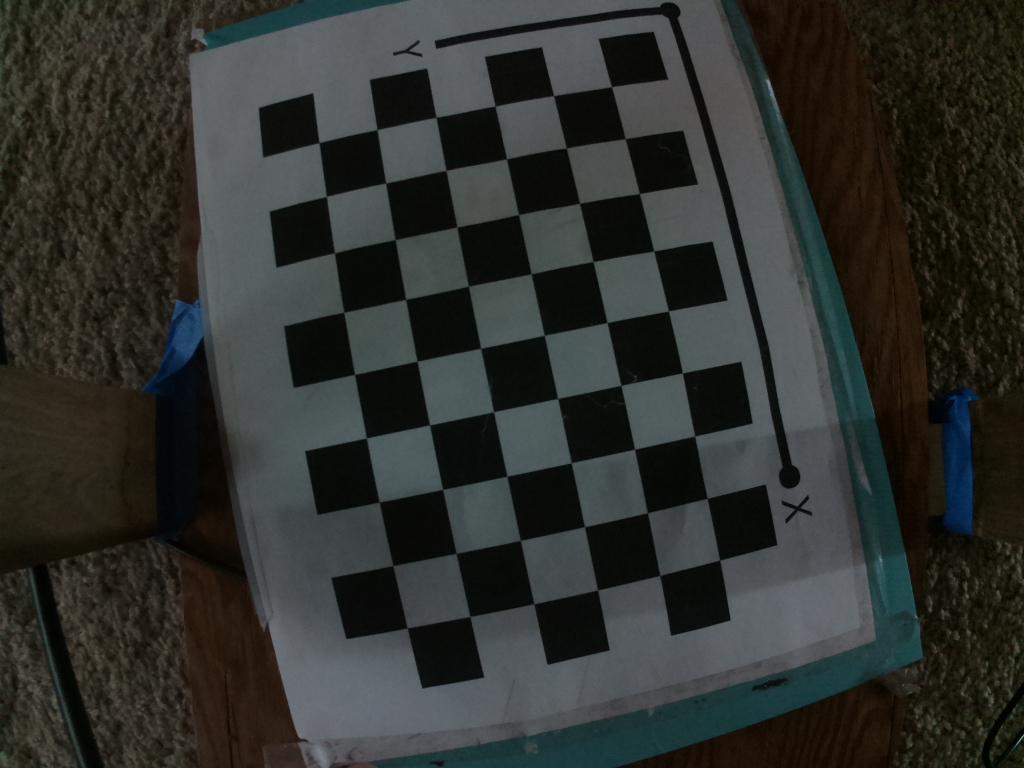

Supplement: Supplementary file 1 [file sensors-23-00560-s001.zip › Combined Data/Calibration/Calibration picam/3 (2).jpeg]

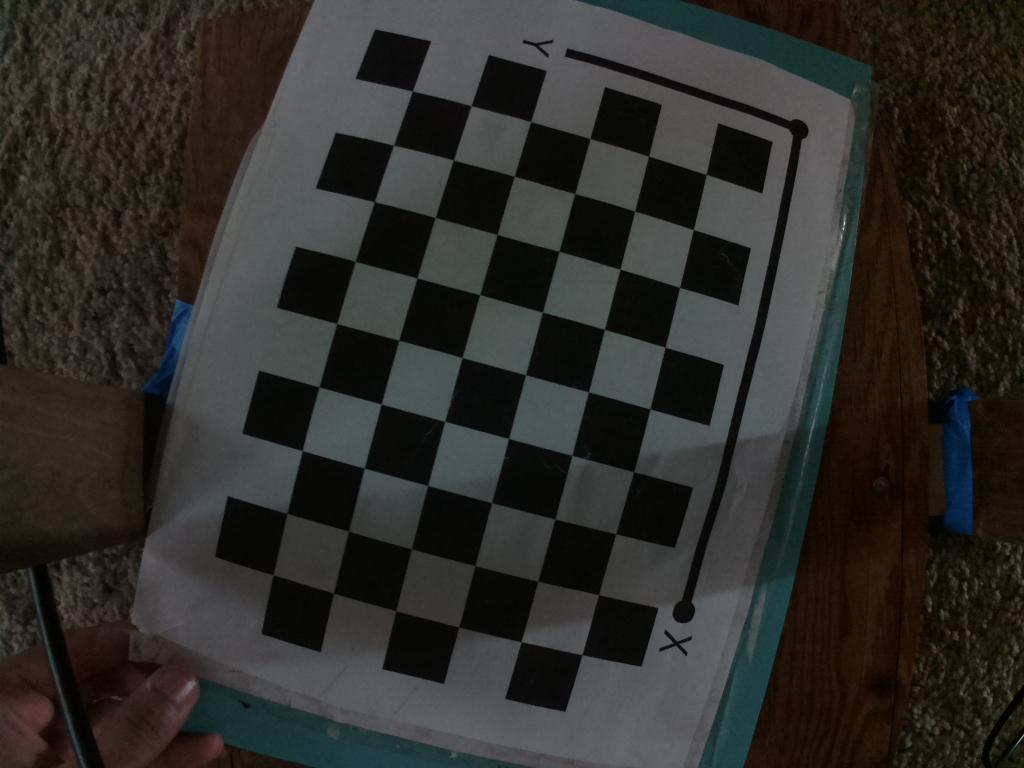

Supplement: Supplementary file 1 [file sensors-23-00560-s001.zip › Combined Data/Calibration/Calibration picam/3 (3).jpeg]

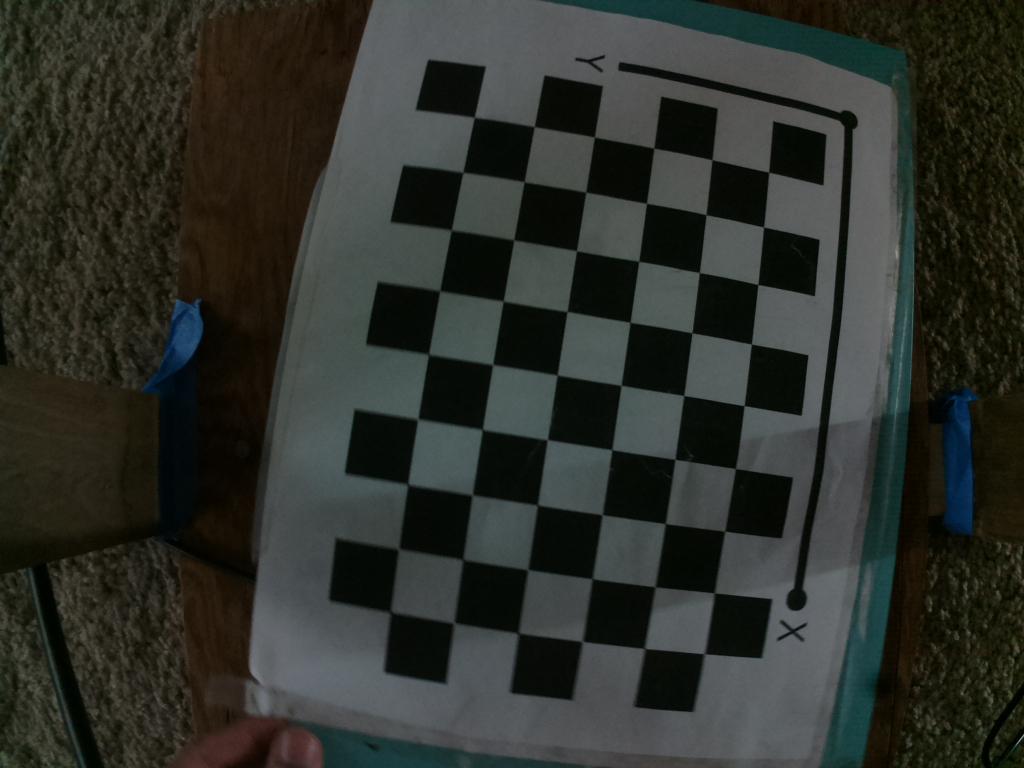

Supplement: Supplementary file 1 [file sensors-23-00560-s001.zip › Combined Data/Calibration/Calibration picam/3 (4).jpeg]

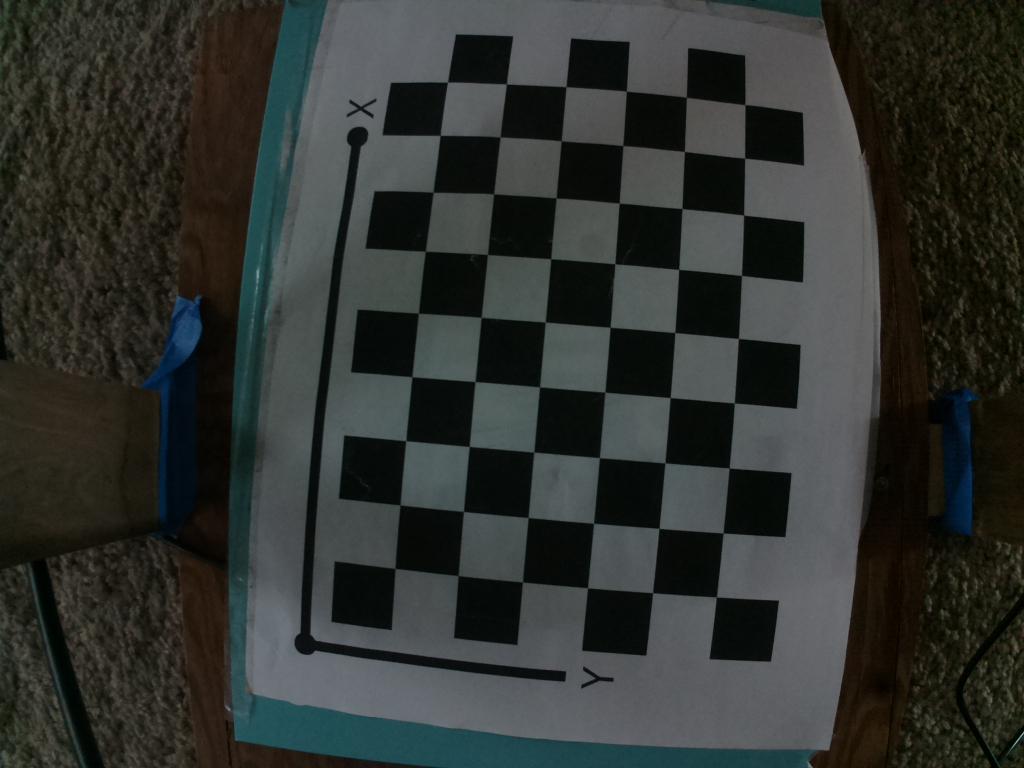

Supplement: Supplementary file 1 [file sensors-23-00560-s001.zip › Combined Data/Calibration/Calibration picam/3 (5).jpeg]

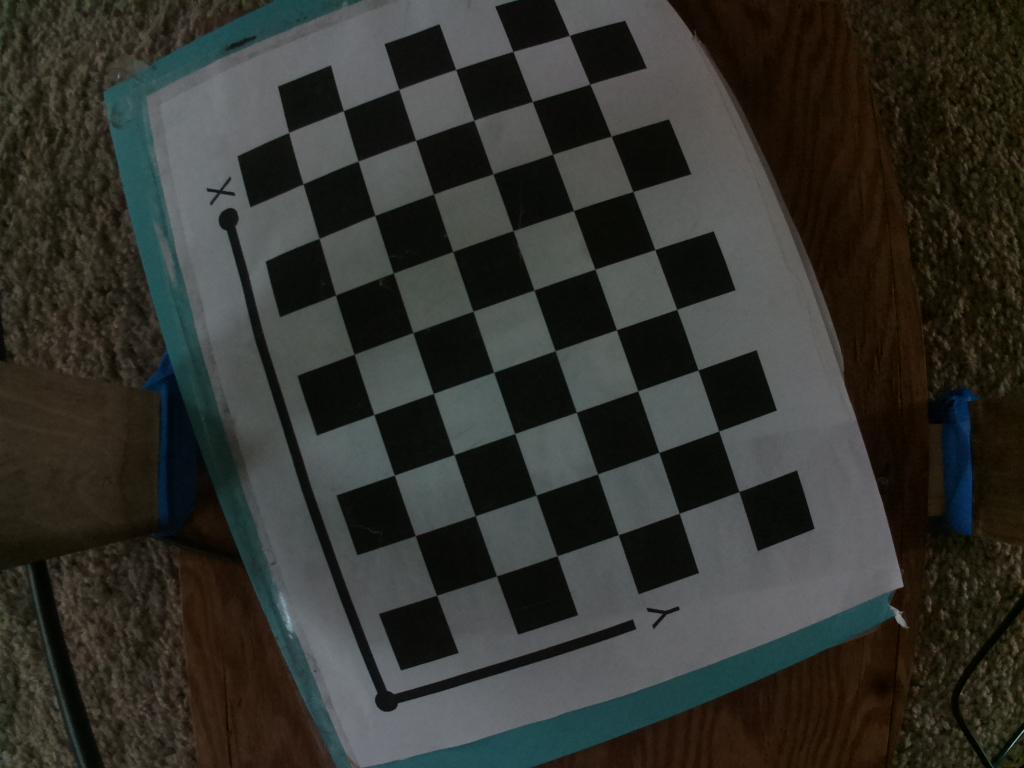

Supplement: Supplementary file 1 [file sensors-23-00560-s001.zip › Combined Data/Calibration/Calibration picam/3 (6).jpeg]

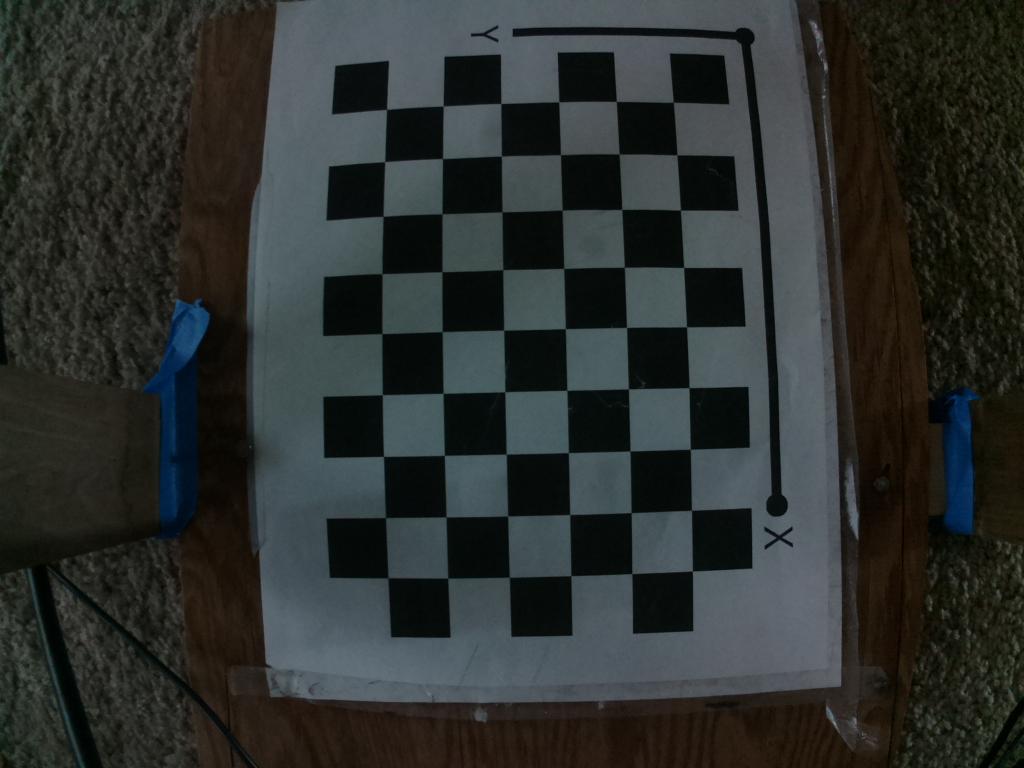

Supplement: Supplementary file 1 [file sensors-23-00560-s001.zip › Combined Data/Calibration/Calibration picam/3.jpeg]

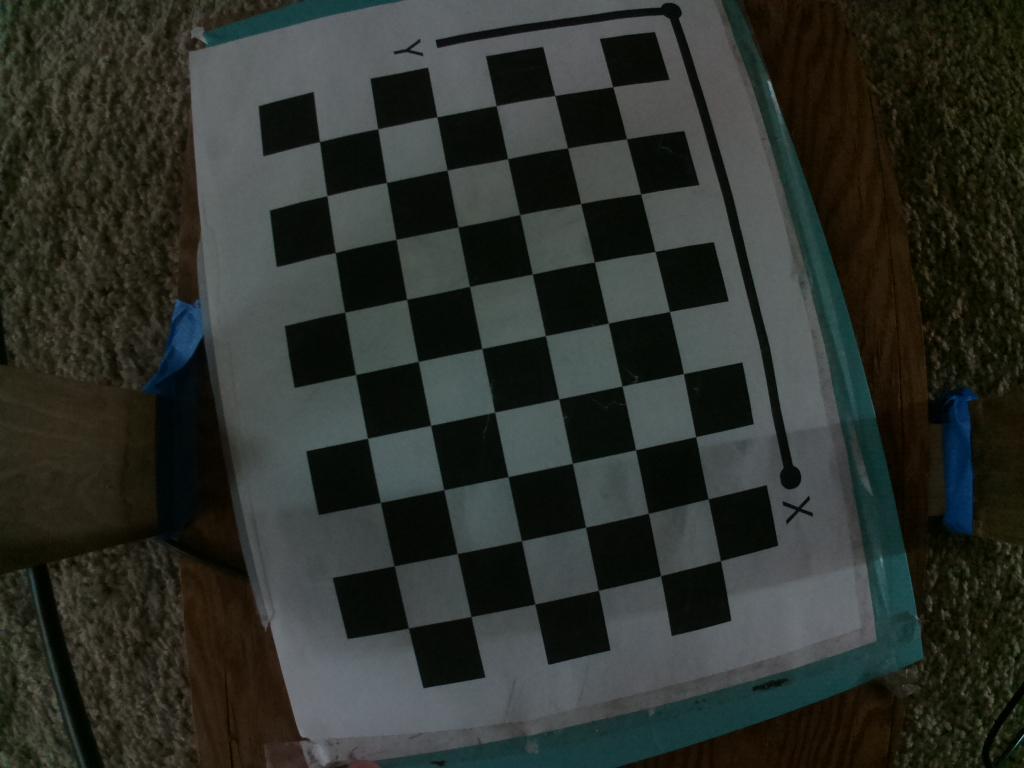

Supplement: Supplementary file 1 [file sensors-23-00560-s001.zip › Combined Data/Calibration/Calibration picam/4 (2).jpeg]

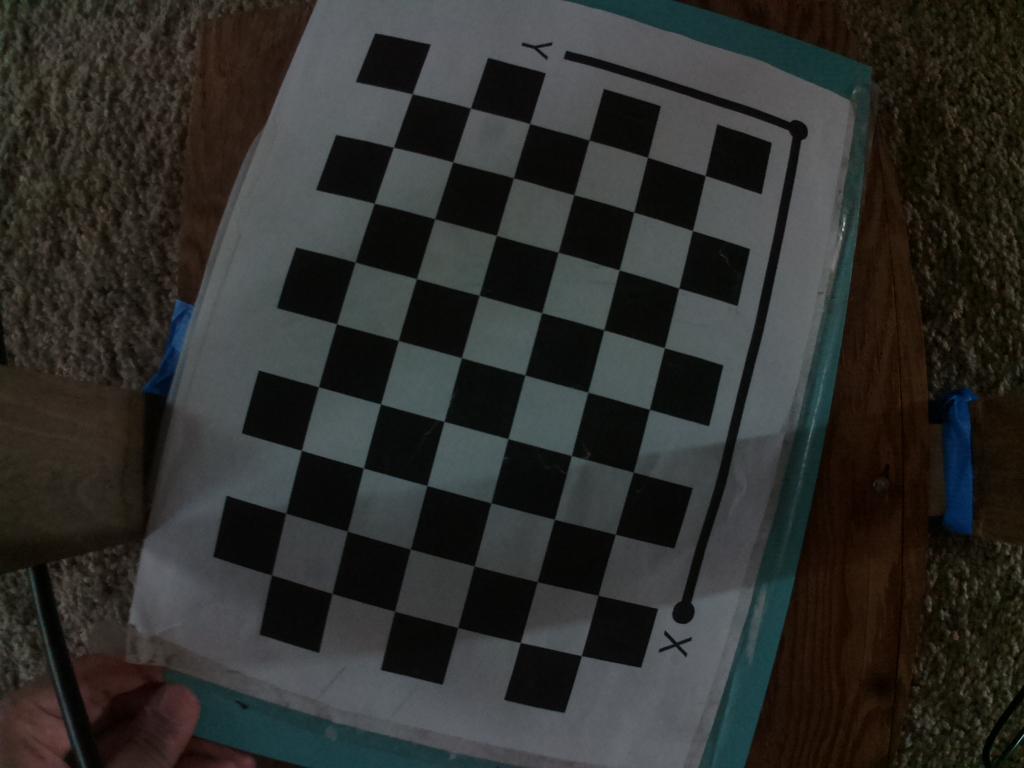

Supplement: Supplementary file 1 [file sensors-23-00560-s001.zip › Combined Data/Calibration/Calibration picam/4 (3).jpeg]

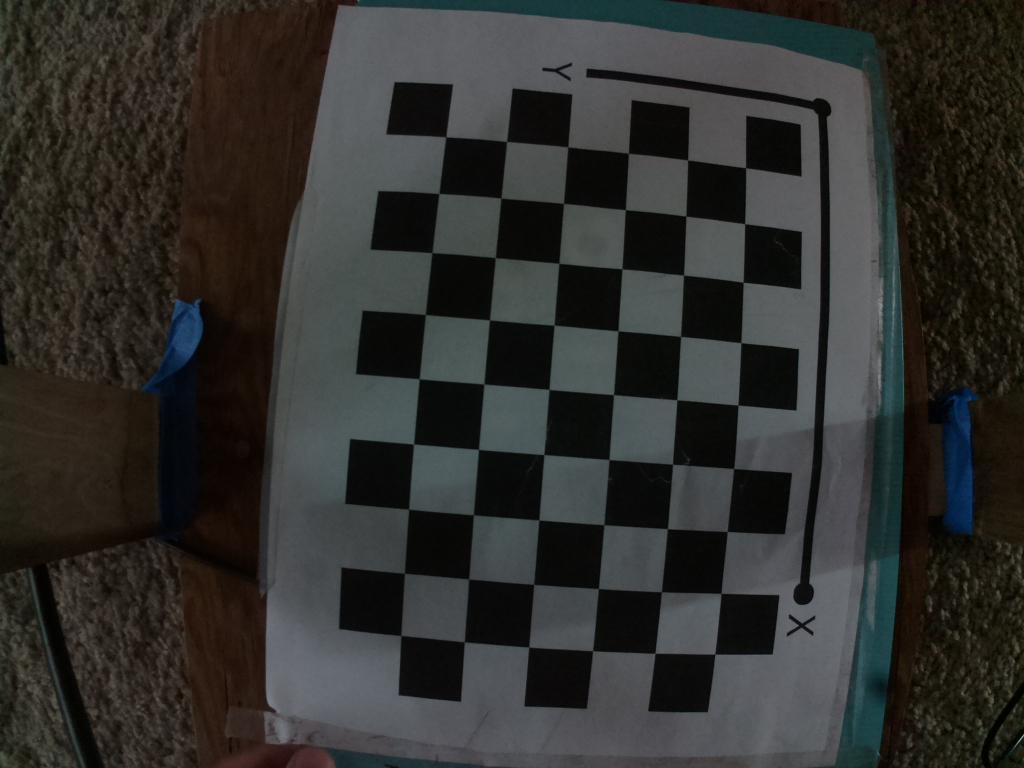

Supplement: Supplementary file 1 [file sensors-23-00560-s001.zip › Combined Data/Calibration/Calibration picam/4 (4).jpeg]

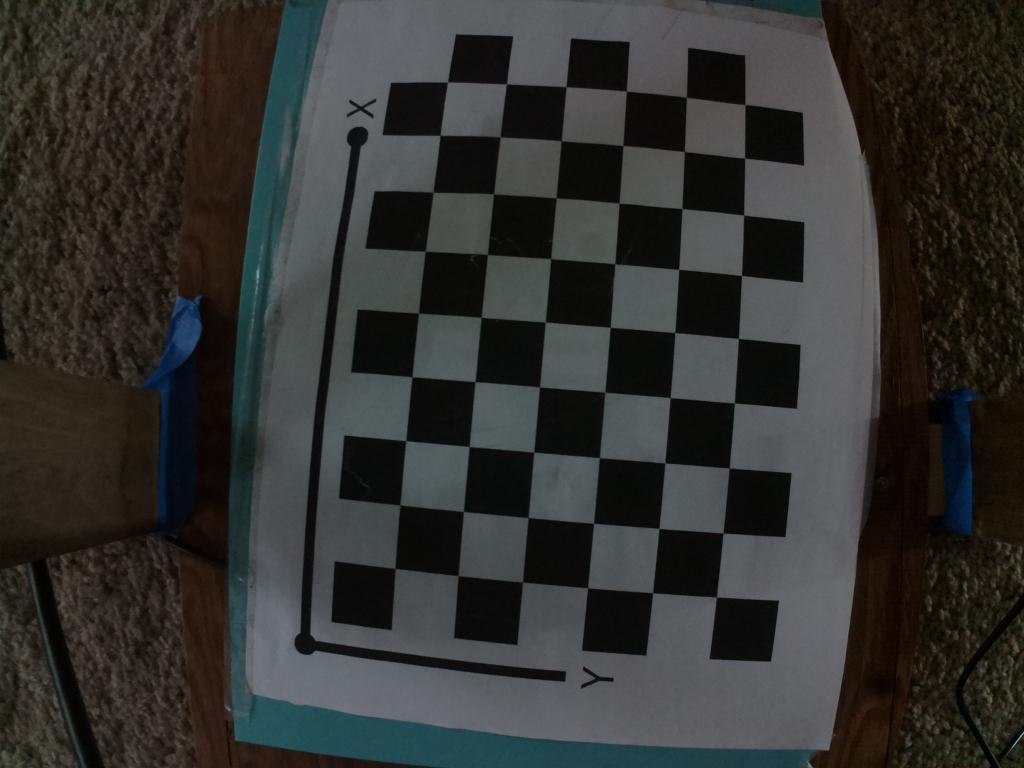

Supplement: Supplementary file 1 [file sensors-23-00560-s001.zip › Combined Data/Calibration/Calibration picam/4 (5).jpeg]

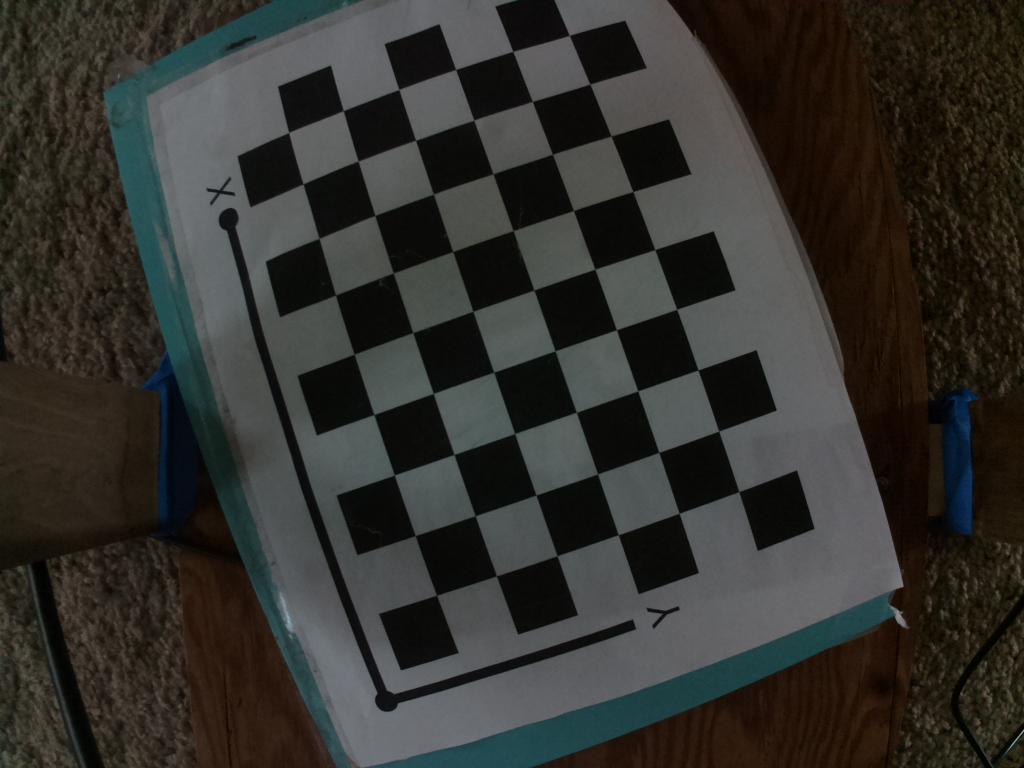

Supplement: Supplementary file 1 [file sensors-23-00560-s001.zip › Combined Data/Calibration/Calibration picam/4 (6).jpeg]

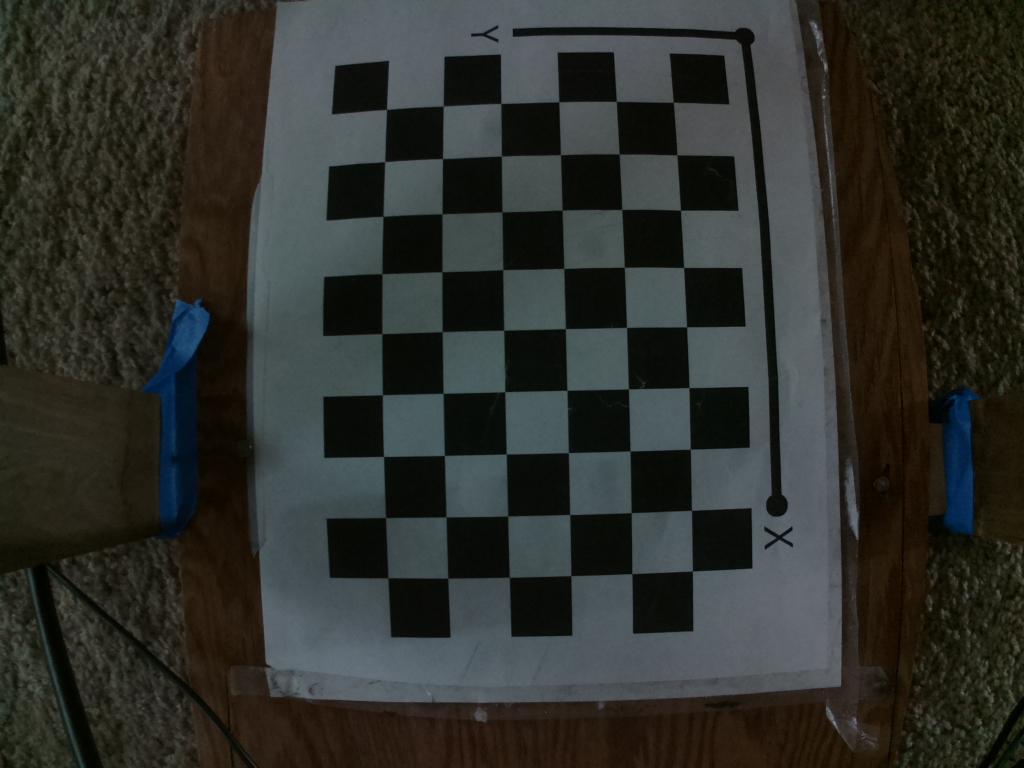

Supplement: Supplementary file 1 [file sensors-23-00560-s001.zip › Combined Data/Calibration/Calibration picam/4.jpeg]

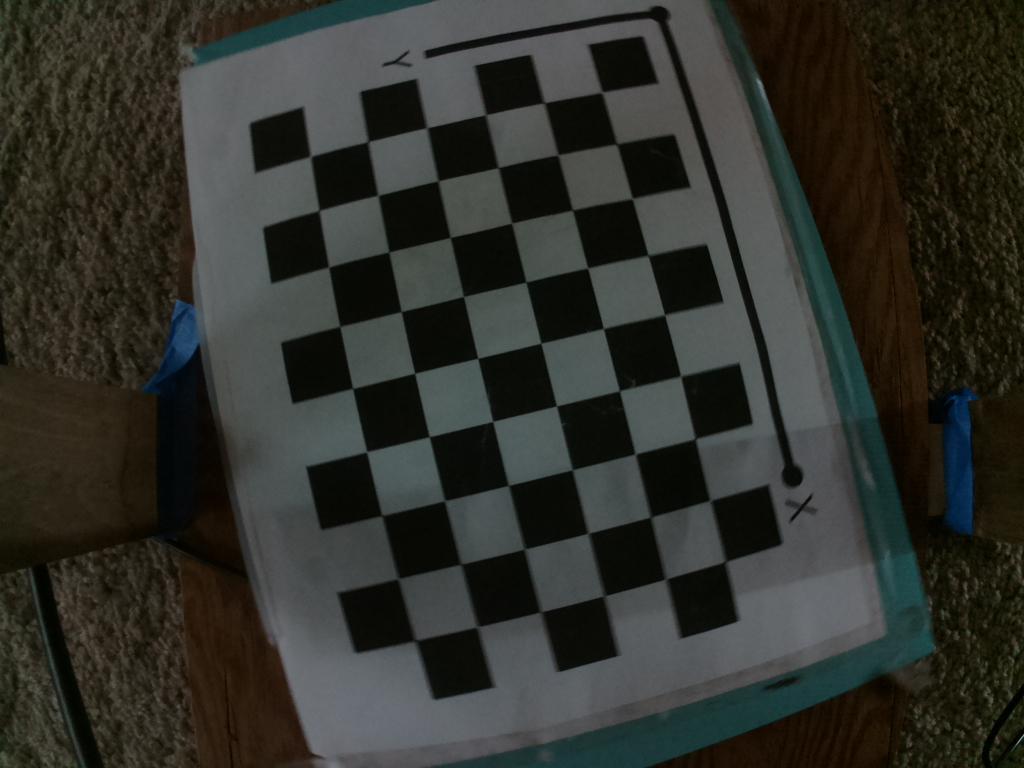

Supplement: Supplementary file 1 [file sensors-23-00560-s001.zip › Combined Data/Calibration/Calibration picam/5 (2).jpeg]

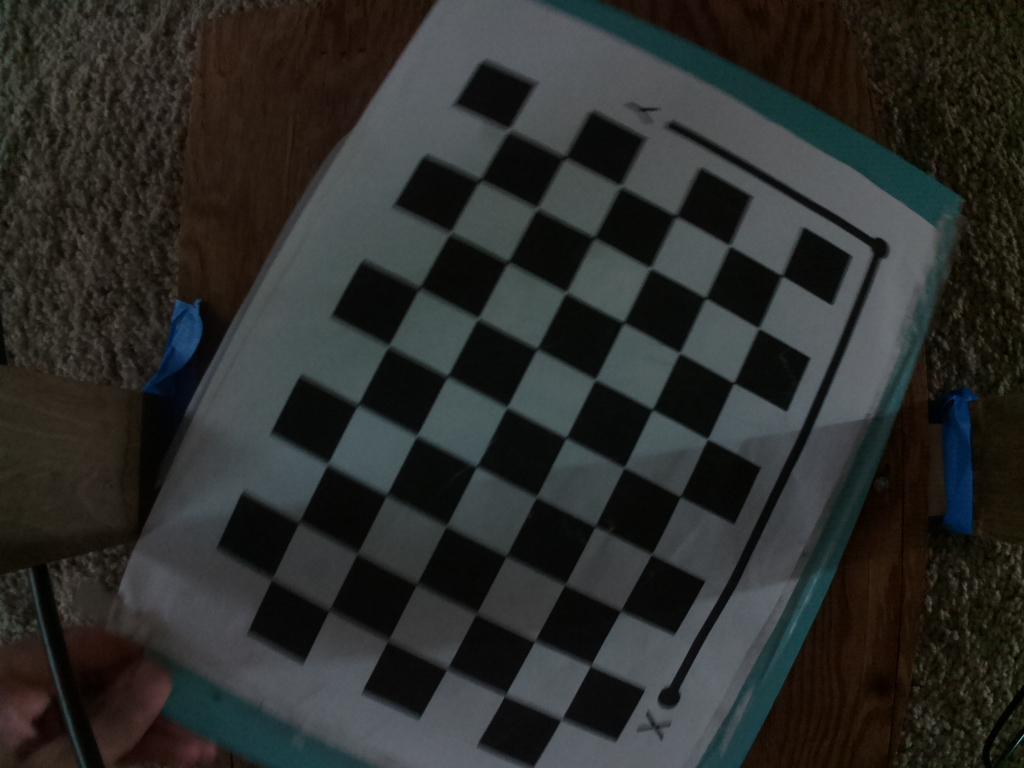

Supplement: Supplementary file 1 [file sensors-23-00560-s001.zip › Combined Data/Calibration/Calibration picam/5 (3).jpeg]

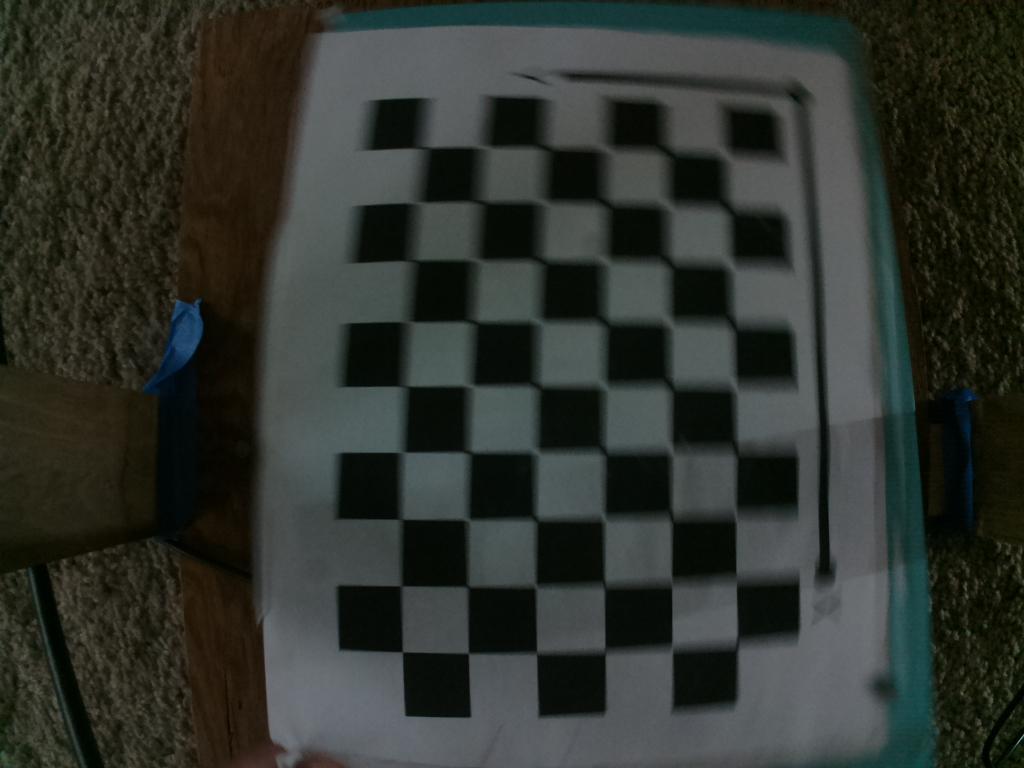

Supplement: Supplementary file 1 [file sensors-23-00560-s001.zip › Combined Data/Calibration/Calibration picam/5 (4).jpeg]

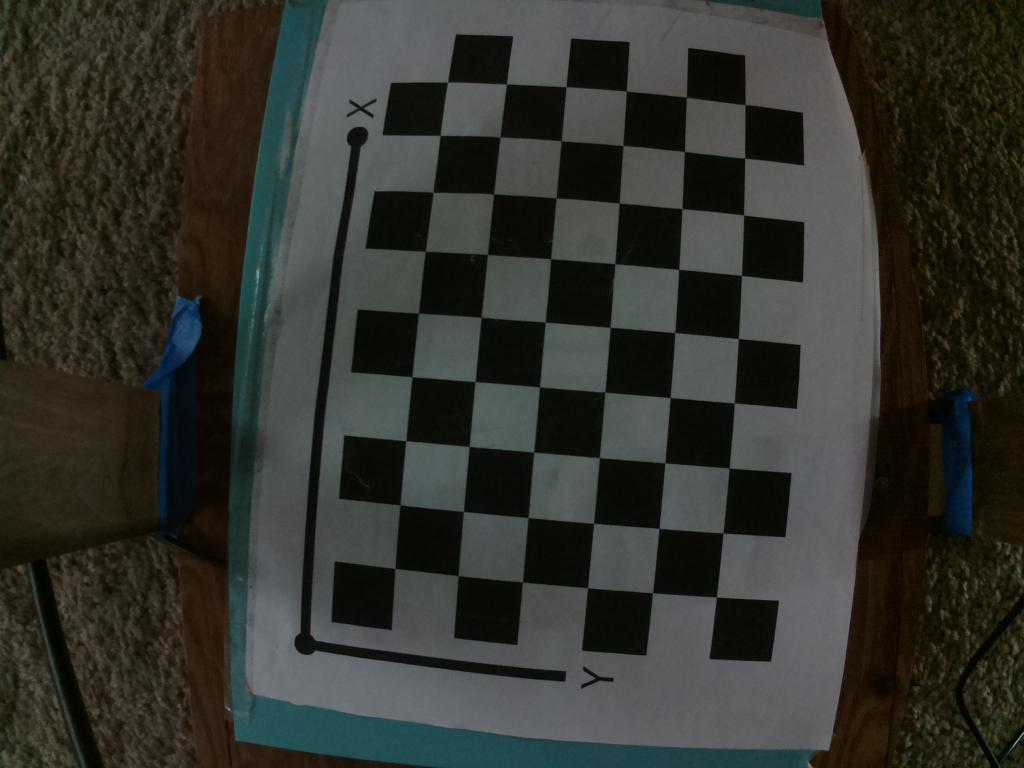

Supplement: Supplementary file 1 [file sensors-23-00560-s001.zip › Combined Data/Calibration/Calibration picam/5 (5).jpeg]

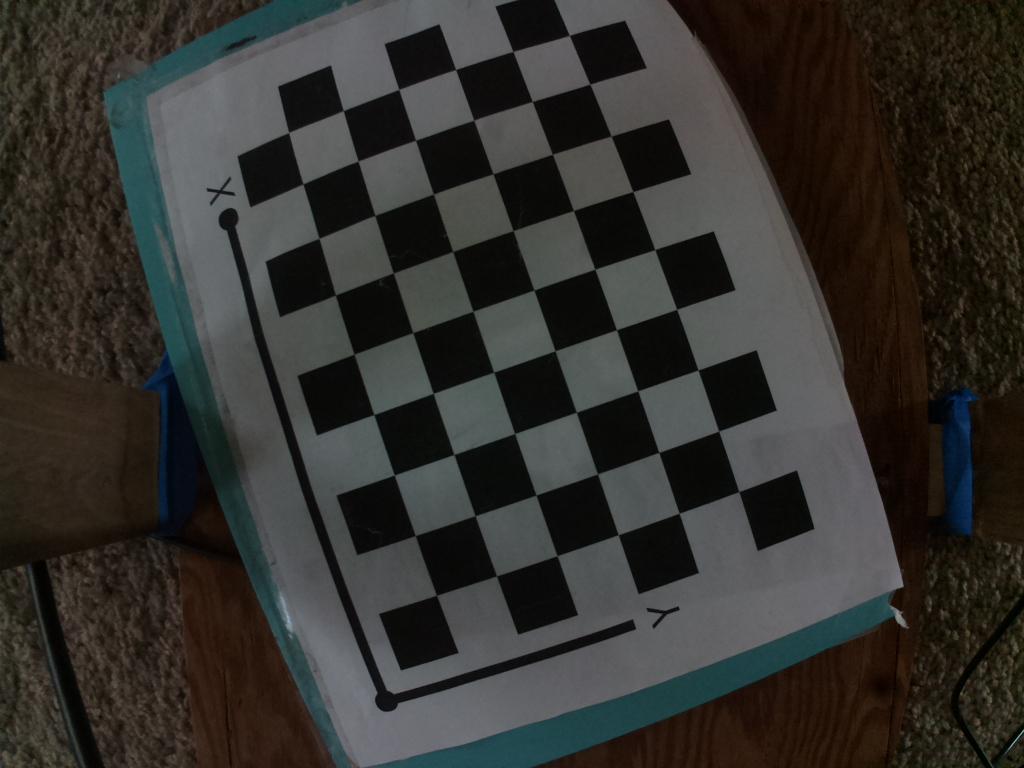

Supplement: Supplementary file 1 [file sensors-23-00560-s001.zip › Combined Data/Calibration/Calibration picam/5 (6).jpeg]

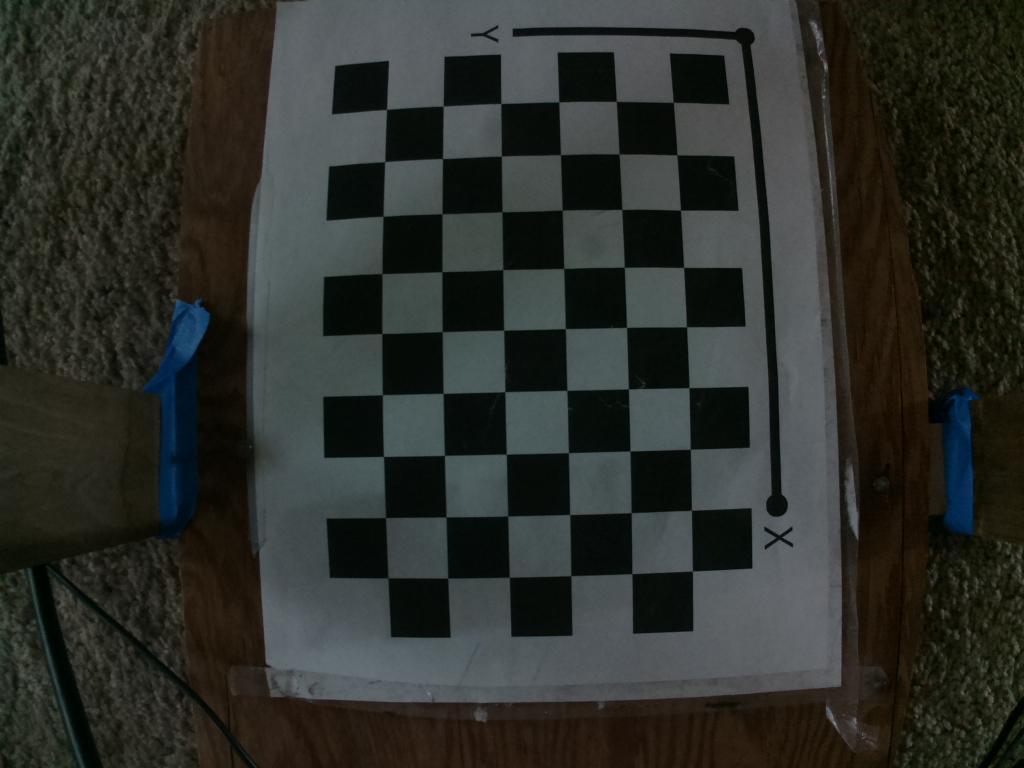

Supplement: Supplementary file 1 [file sensors-23-00560-s001.zip › Combined Data/Calibration/Calibration picam/5.jpeg]

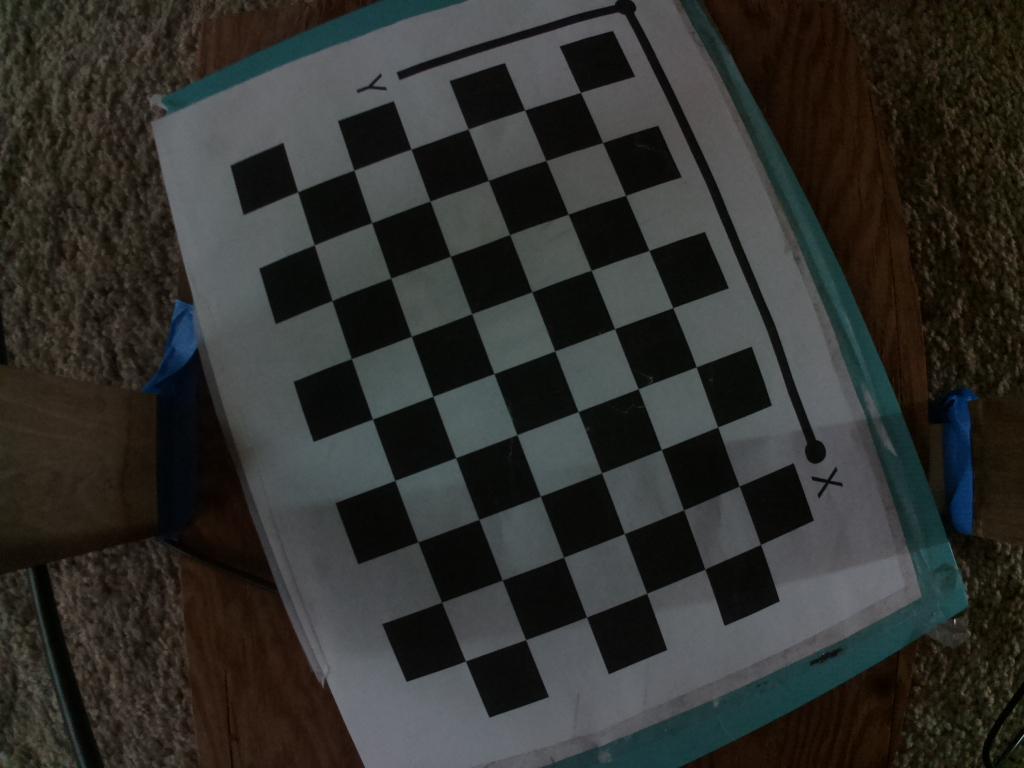

Supplement: Supplementary file 1 [file sensors-23-00560-s001.zip › Combined Data/Calibration/Calibration picam/6 (2).jpeg]

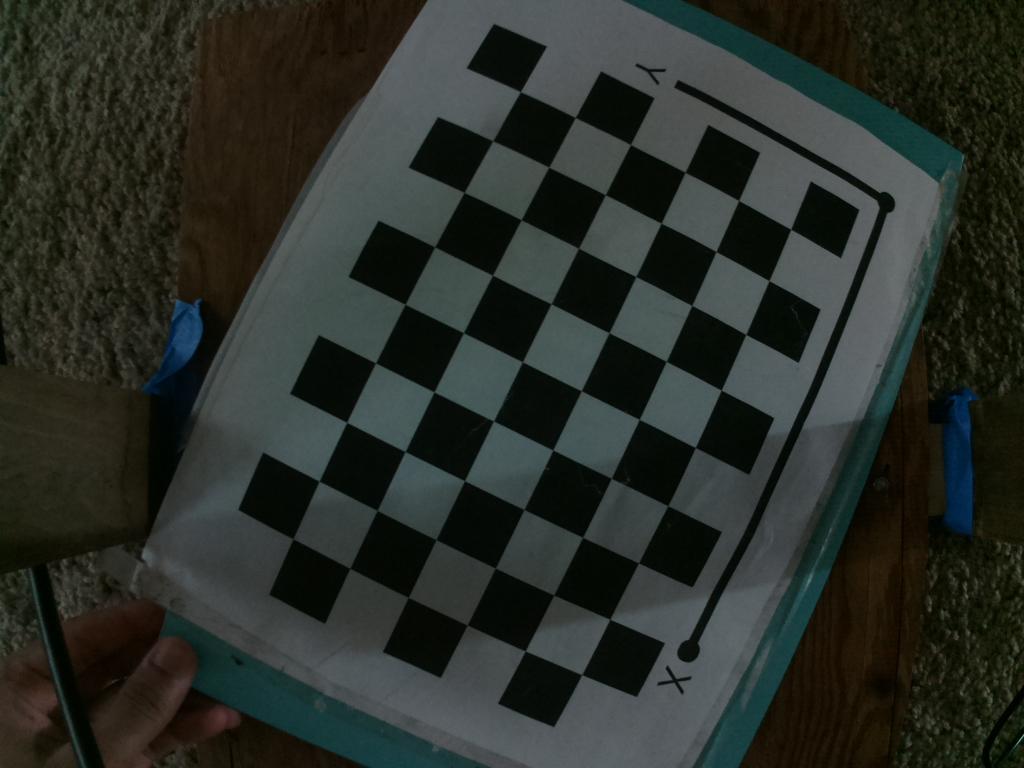

Supplement: Supplementary file 1 [file sensors-23-00560-s001.zip › Combined Data/Calibration/Calibration picam/6 (3).jpeg]

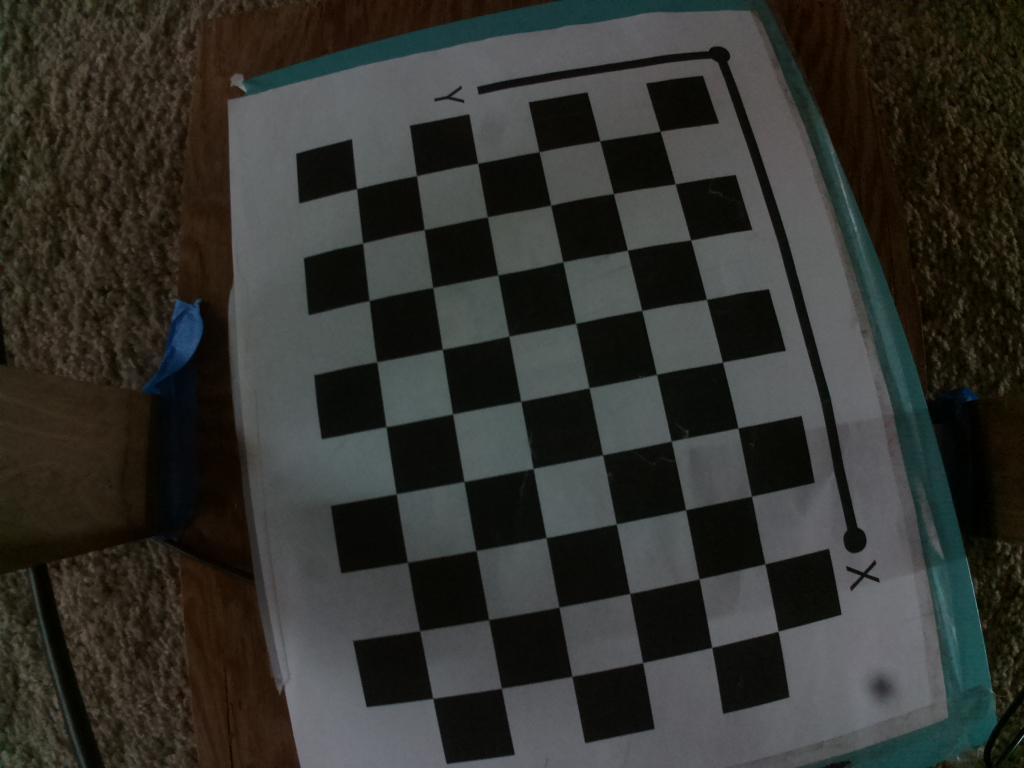

Supplement: Supplementary file 1 [file sensors-23-00560-s001.zip › Combined Data/Calibration/Calibration picam/6 (4).jpeg]

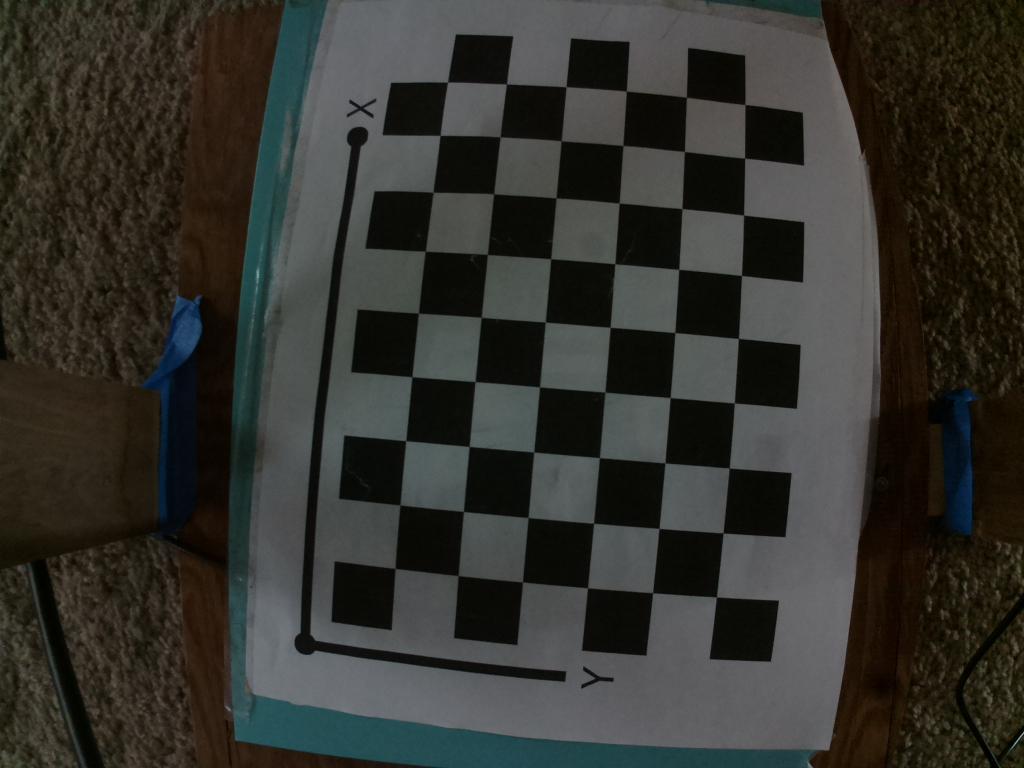

Supplement: Supplementary file 1 [file sensors-23-00560-s001.zip › Combined Data/Calibration/Calibration picam/6 (5).jpeg]

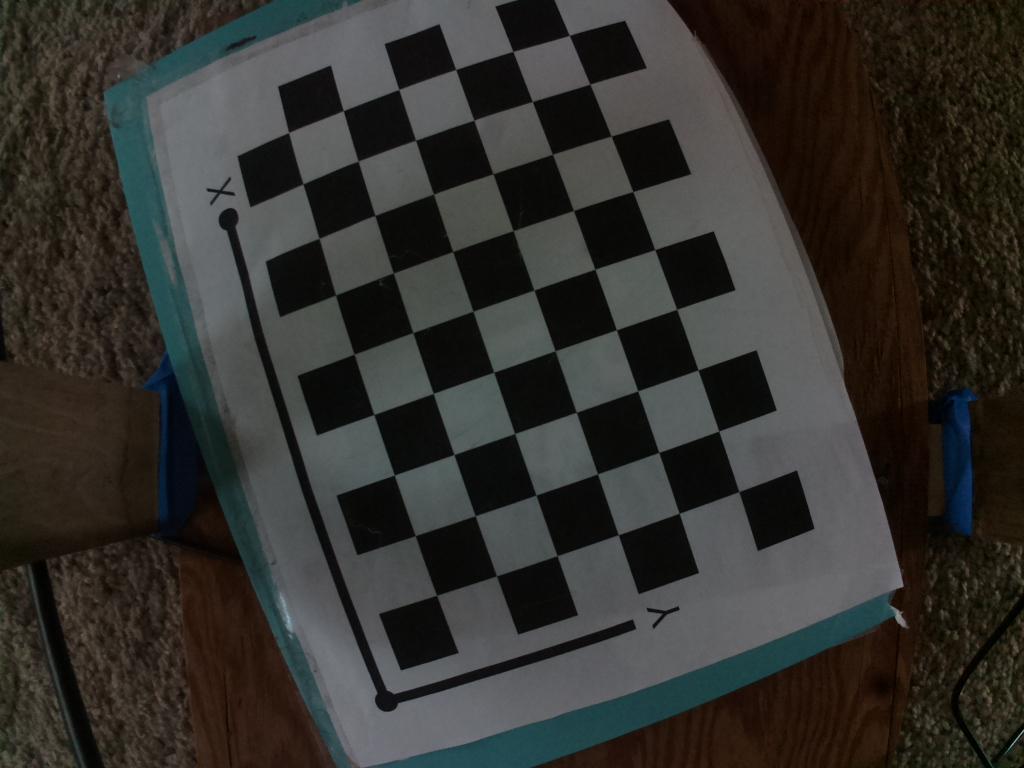

Supplement: Supplementary file 1 [file sensors-23-00560-s001.zip › Combined Data/Calibration/Calibration picam/6 (6).jpeg]

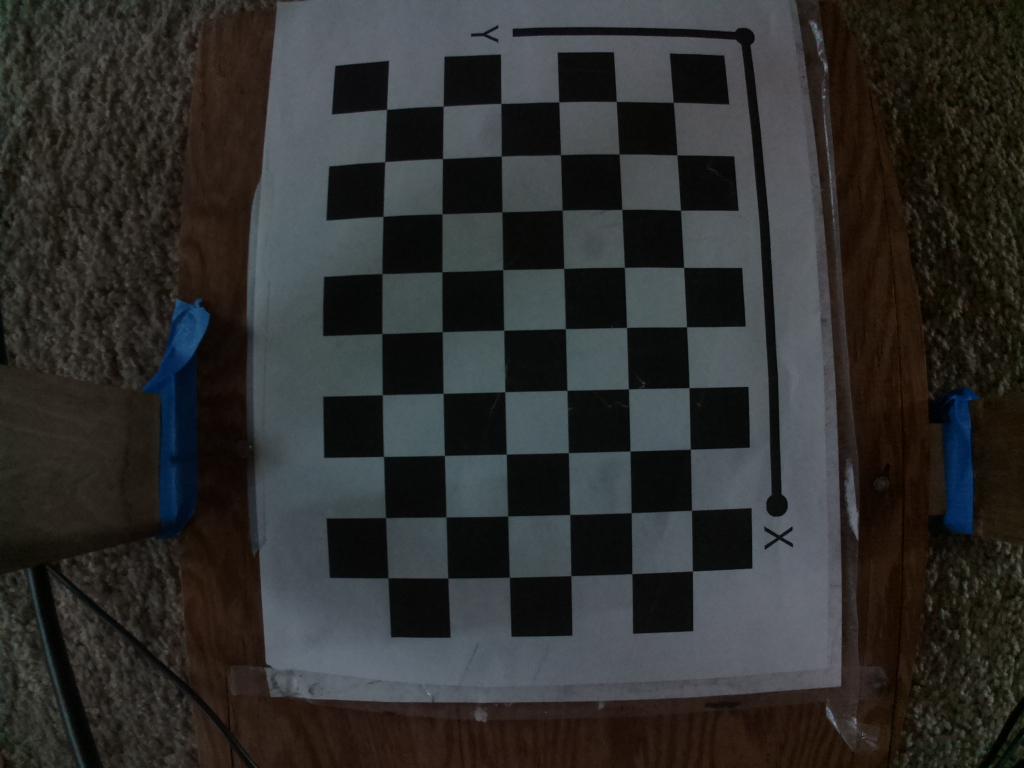

Supplement: Supplementary file 1 [file sensors-23-00560-s001.zip › Combined Data/Calibration/Calibration picam/6.jpeg]

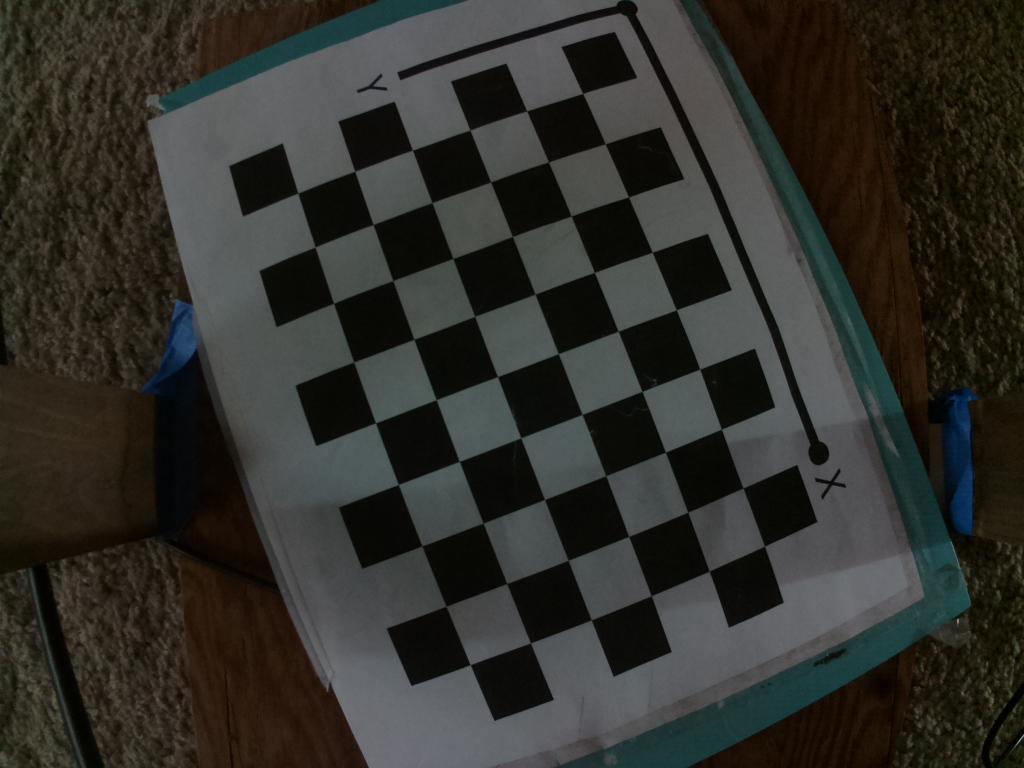

Supplement: Supplementary file 1 [file sensors-23-00560-s001.zip › Combined Data/Calibration/Calibration picam/7 (2).jpeg]

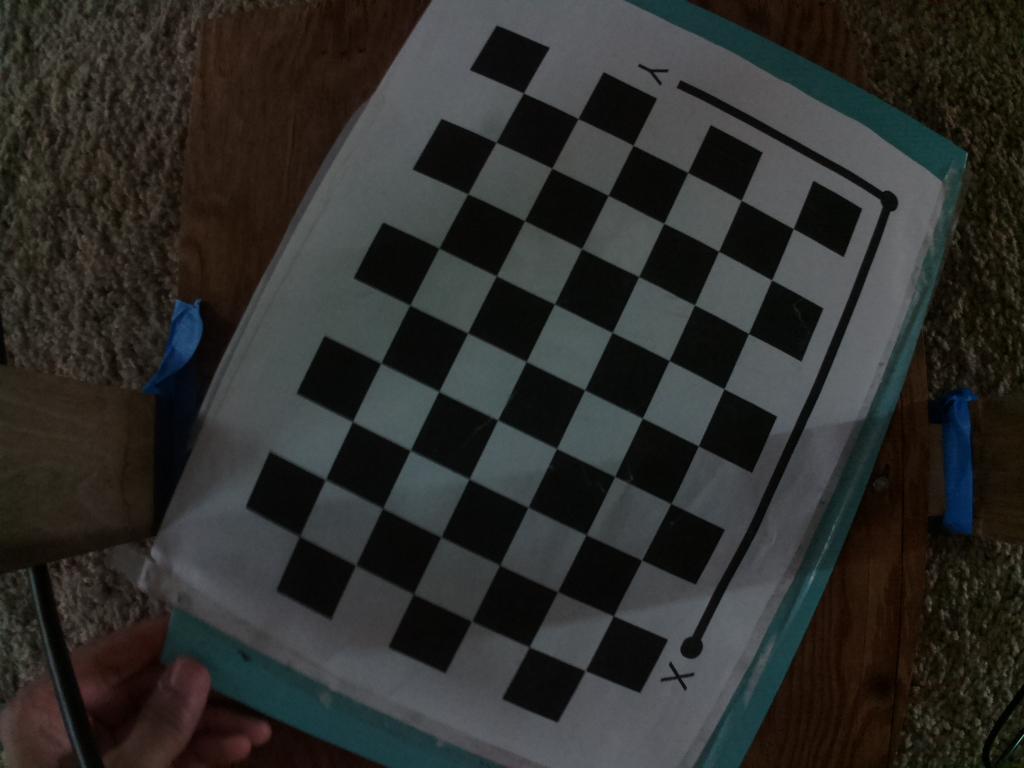

Supplement: Supplementary file 1 [file sensors-23-00560-s001.zip › Combined Data/Calibration/Calibration picam/7 (3).jpeg]

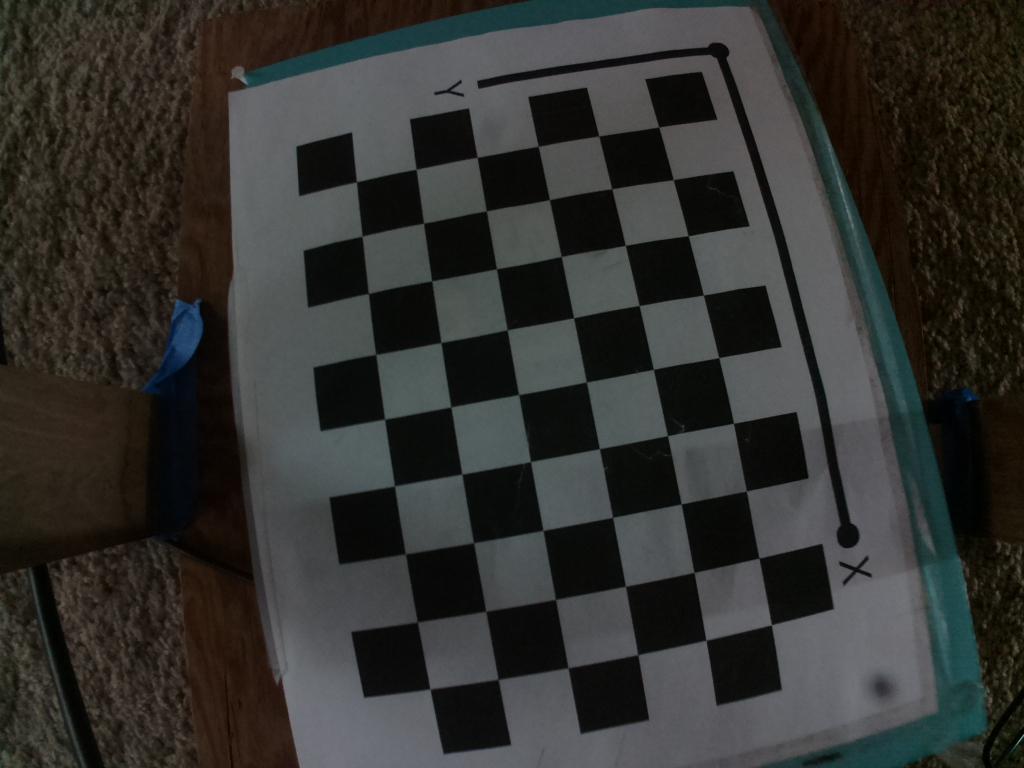

Supplement: Supplementary file 1 [file sensors-23-00560-s001.zip › Combined Data/Calibration/Calibration picam/7 (4).jpeg]

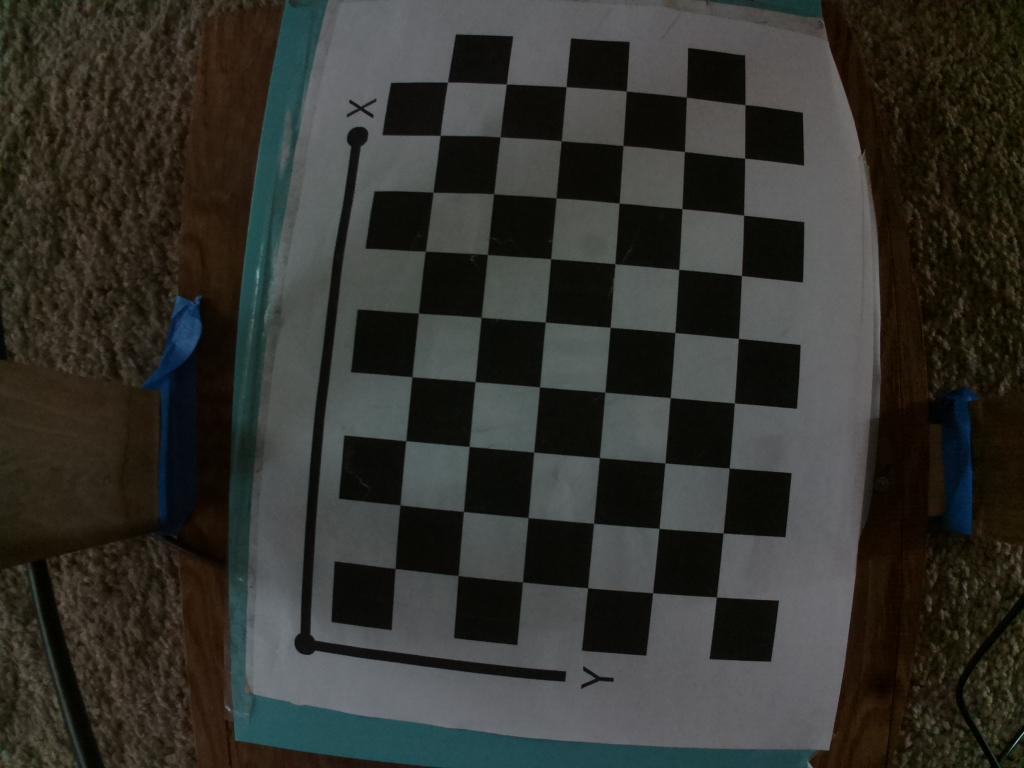

Supplement: Supplementary file 1 [file sensors-23-00560-s001.zip › Combined Data/Calibration/Calibration picam/7 (5).jpeg]

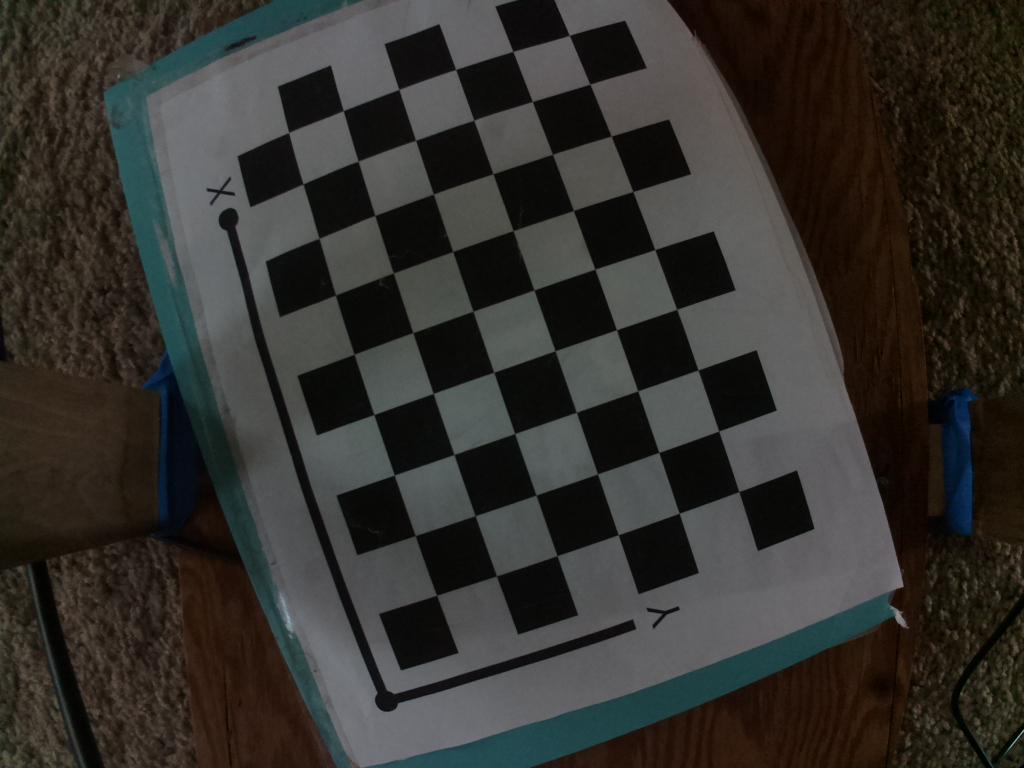

Supplement: Supplementary file 1 [file sensors-23-00560-s001.zip › Combined Data/Calibration/Calibration picam/7 (6).jpeg]

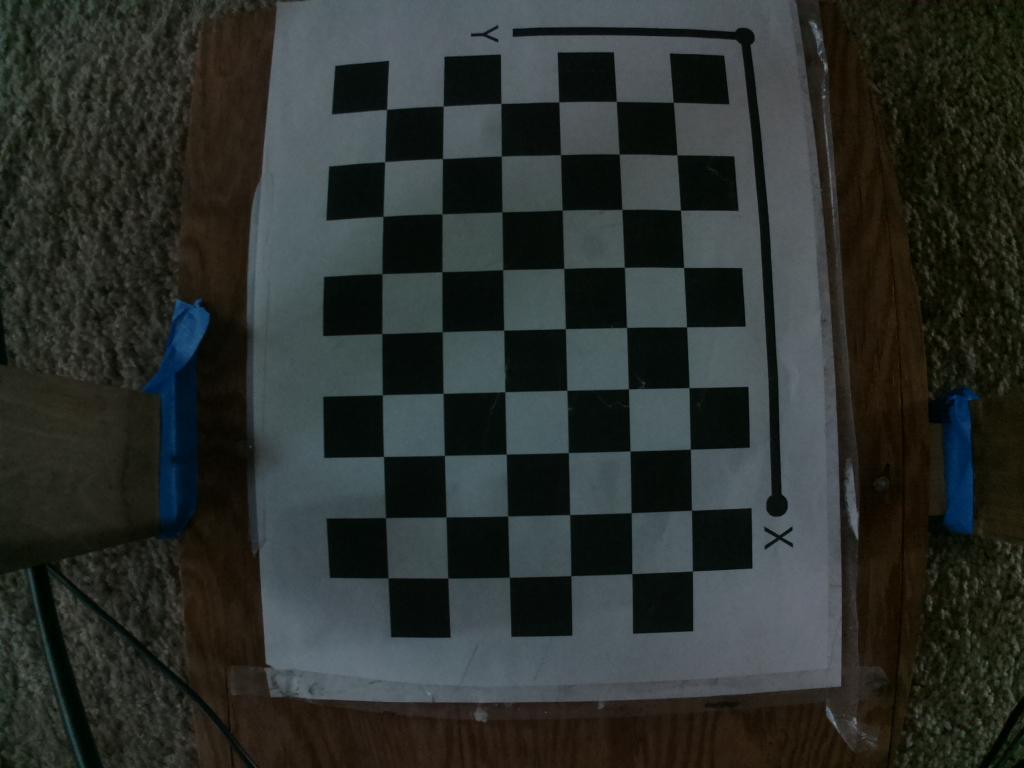

Supplement: Supplementary file 1 [file sensors-23-00560-s001.zip › Combined Data/Calibration/Calibration picam/7.jpeg]

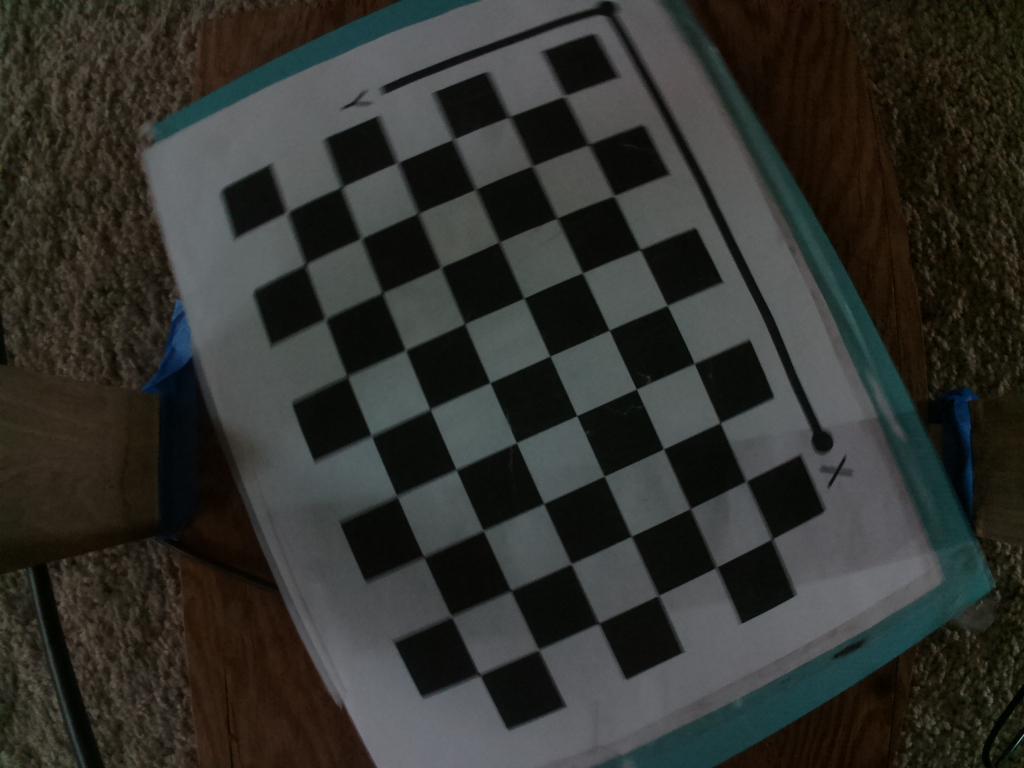

Supplement: Supplementary file 1 [file sensors-23-00560-s001.zip › Combined Data/Calibration/Calibration picam/8 (2).jpeg]

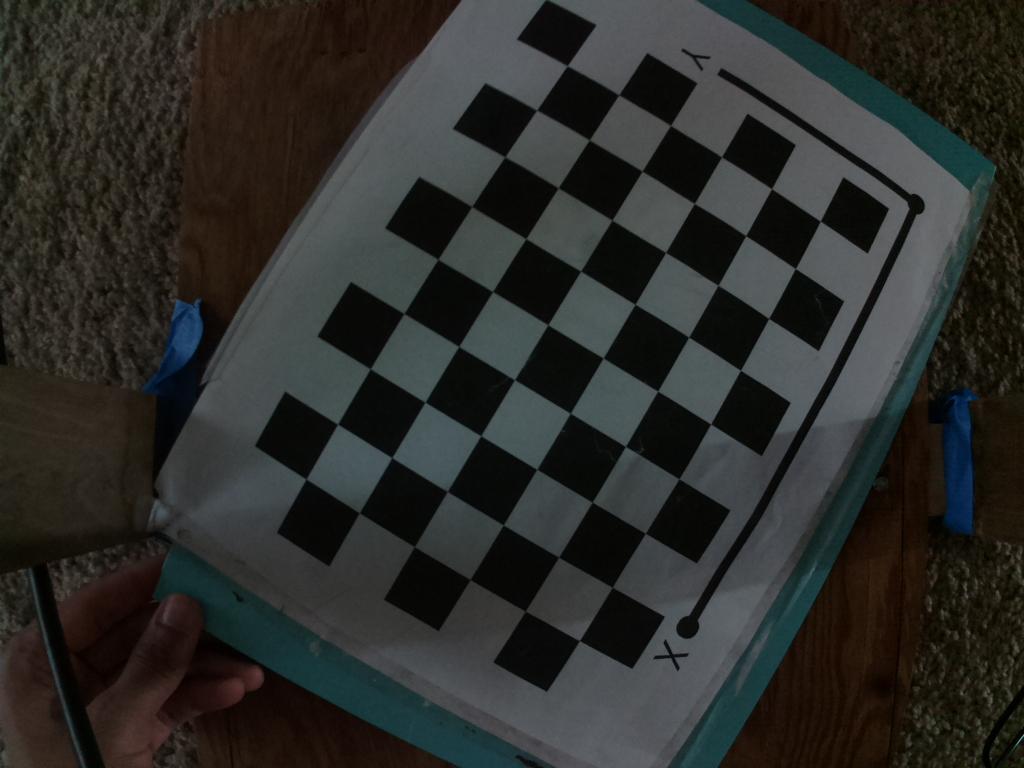

Supplement: Supplementary file 1 [file sensors-23-00560-s001.zip › Combined Data/Calibration/Calibration picam/8 (3).jpeg]

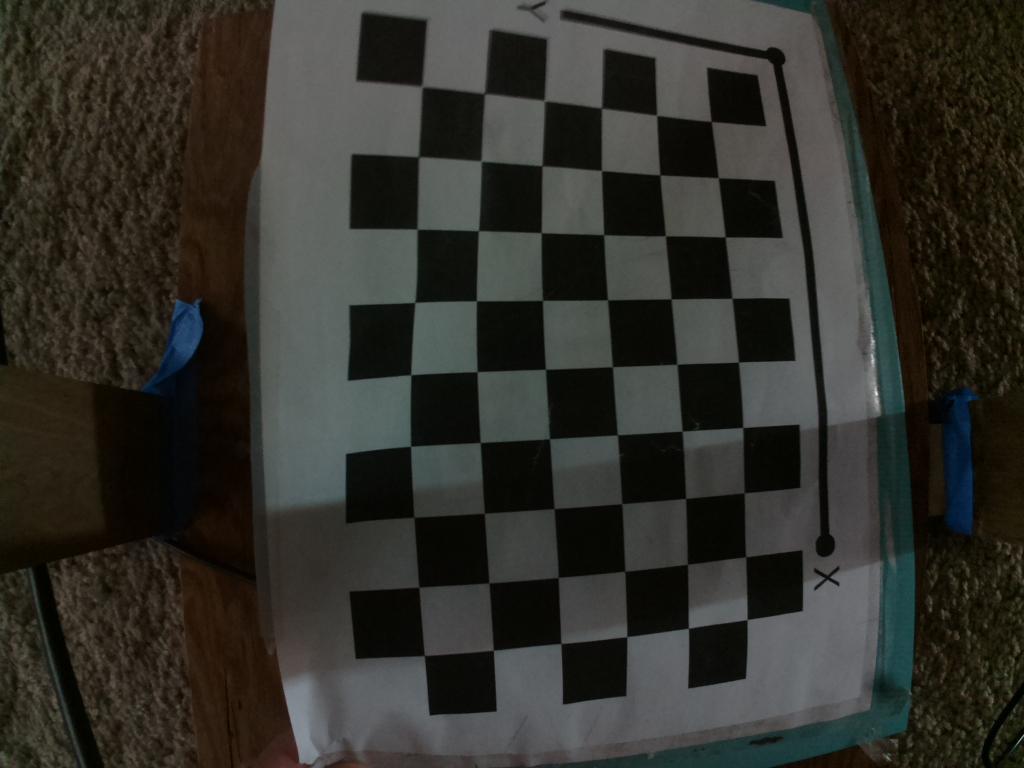

Supplement: Supplementary file 1 [file sensors-23-00560-s001.zip › Combined Data/Calibration/Calibration picam/8 (4).jpeg]

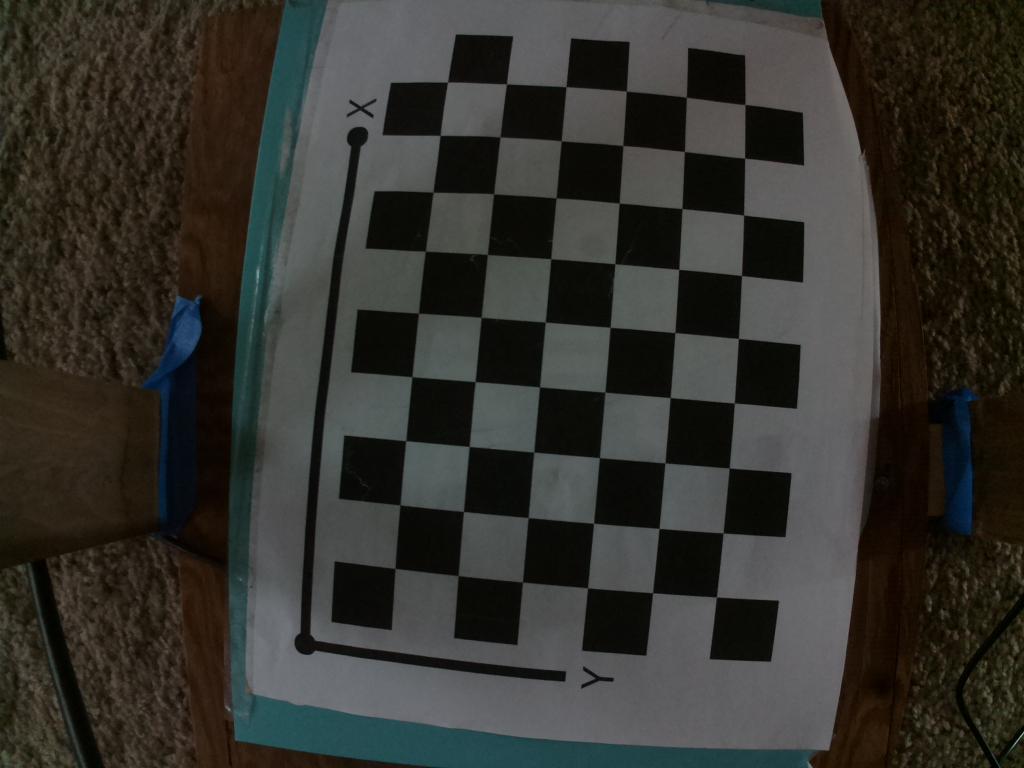

Supplement: Supplementary file 1 [file sensors-23-00560-s001.zip › Combined Data/Calibration/Calibration picam/8 (5).jpeg]

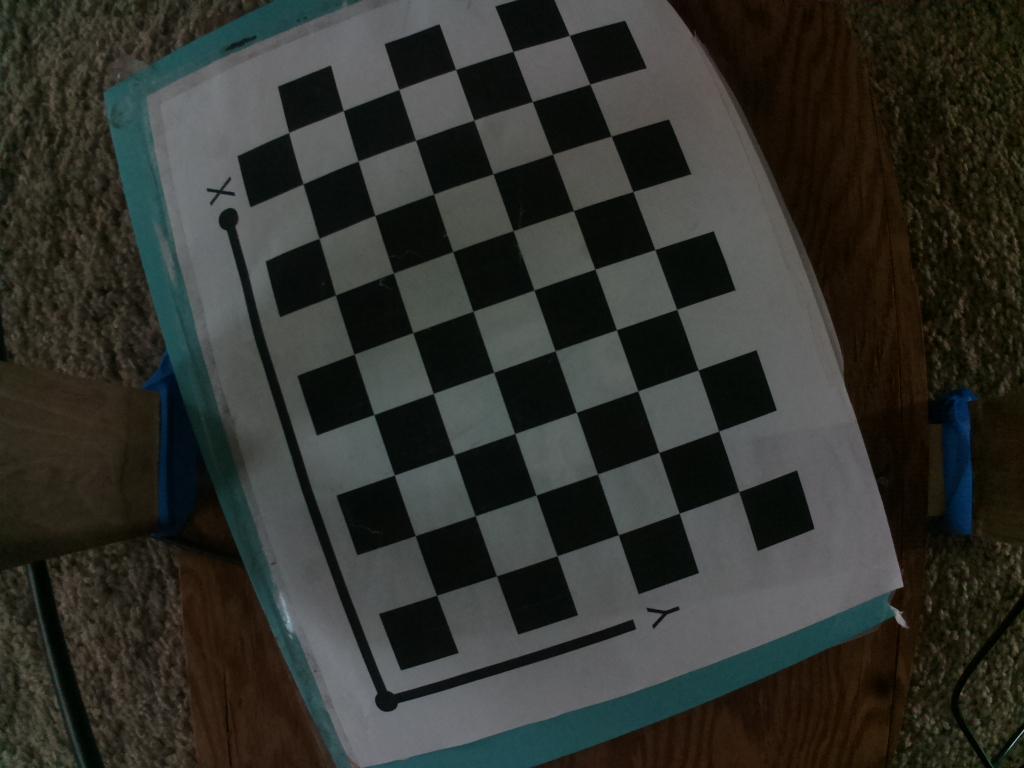

Supplement: Supplementary file 1 [file sensors-23-00560-s001.zip › Combined Data/Calibration/Calibration picam/8 (6).jpeg]

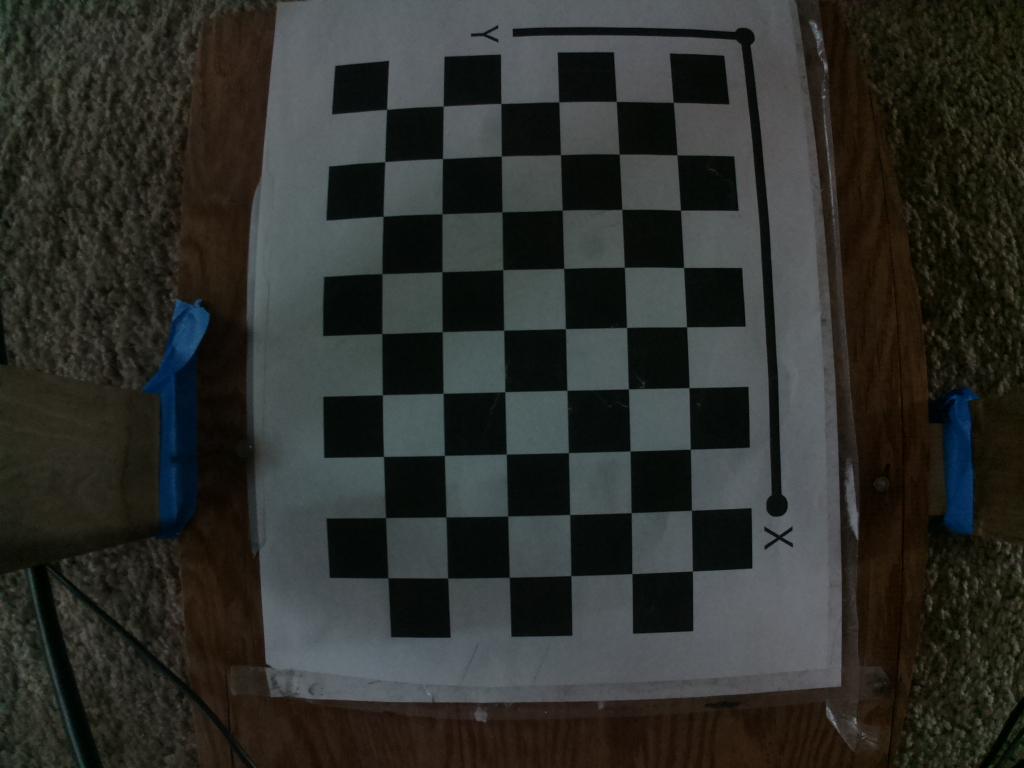

Supplement: Supplementary file 1 [file sensors-23-00560-s001.zip › Combined Data/Calibration/Calibration picam/8.jpeg]

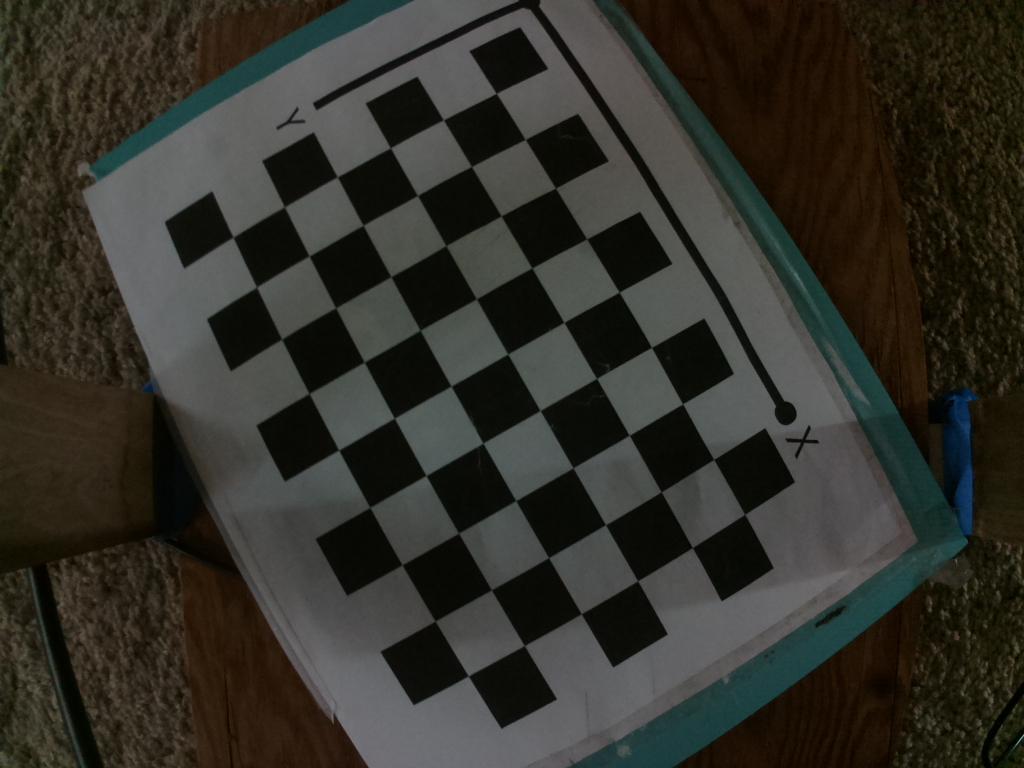

Supplement: Supplementary file 1 [file sensors-23-00560-s001.zip › Combined Data/Calibration/Calibration picam/9 (2).jpeg]

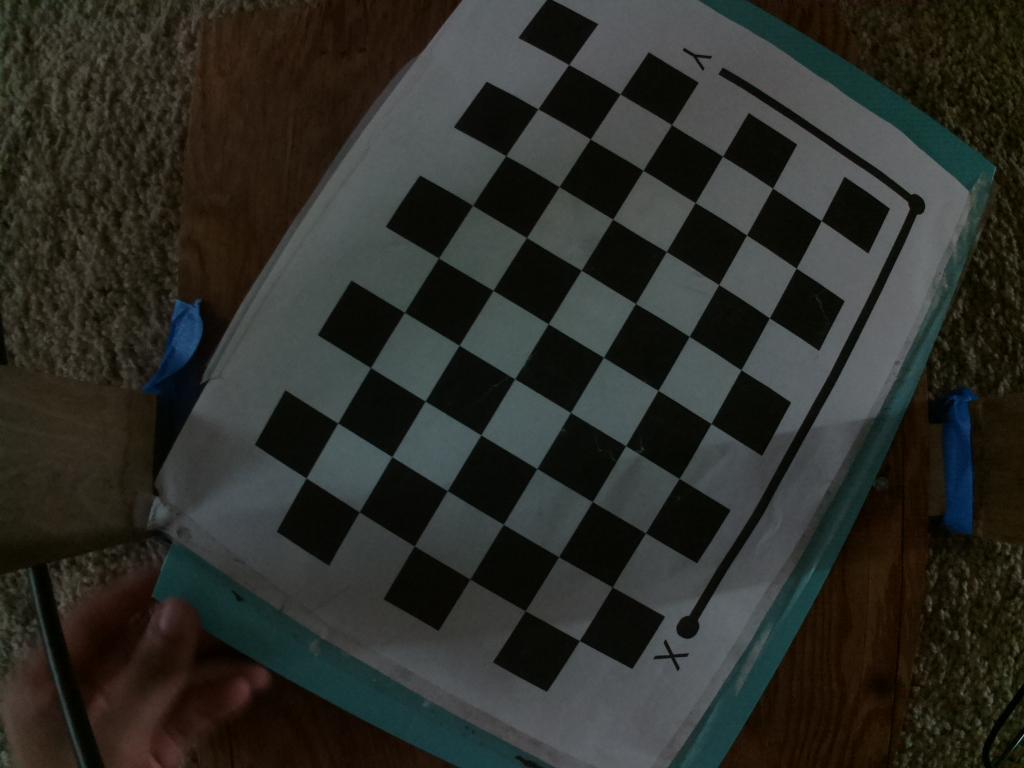

Supplement: Supplementary file 1 [file sensors-23-00560-s001.zip › Combined Data/Calibration/Calibration picam/9 (3).jpeg]

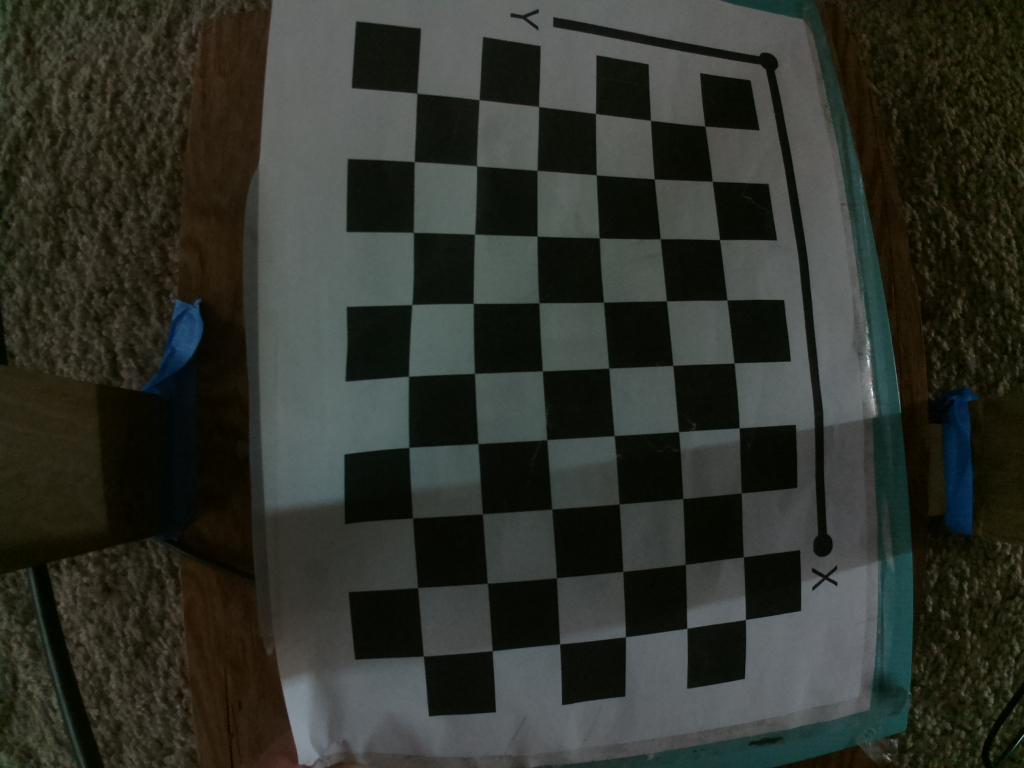

Supplement: Supplementary file 1 [file sensors-23-00560-s001.zip › Combined Data/Calibration/Calibration picam/9 (4).jpeg]

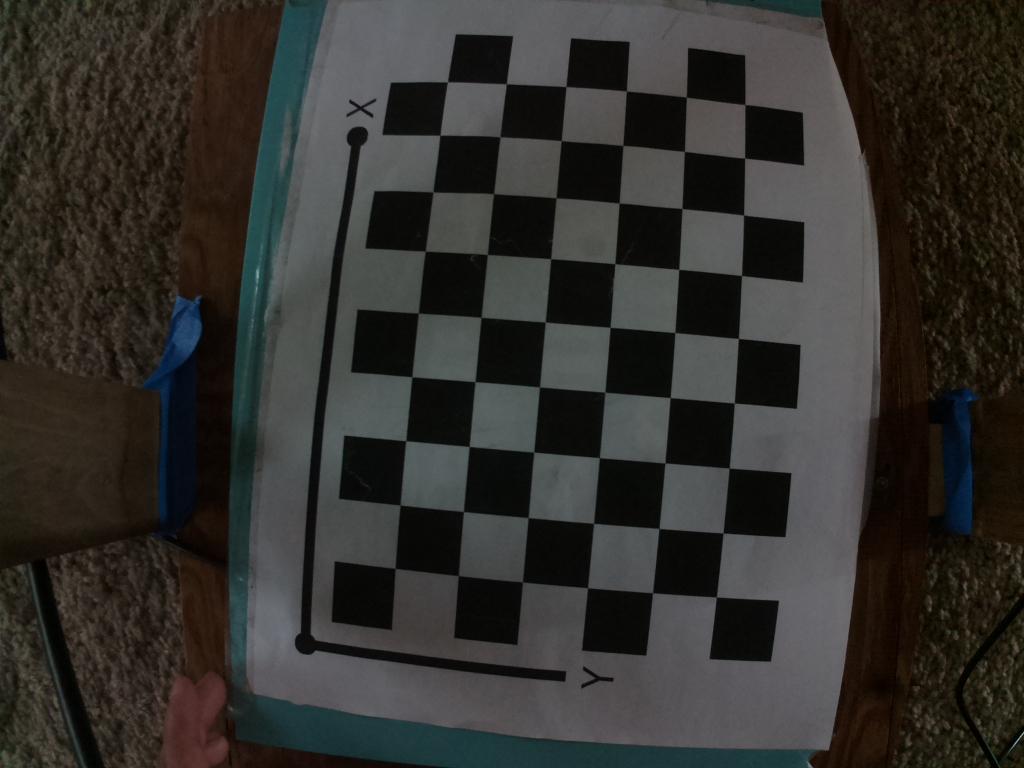

Supplement: Supplementary file 1 [file sensors-23-00560-s001.zip › Combined Data/Calibration/Calibration picam/9 (5).jpeg]

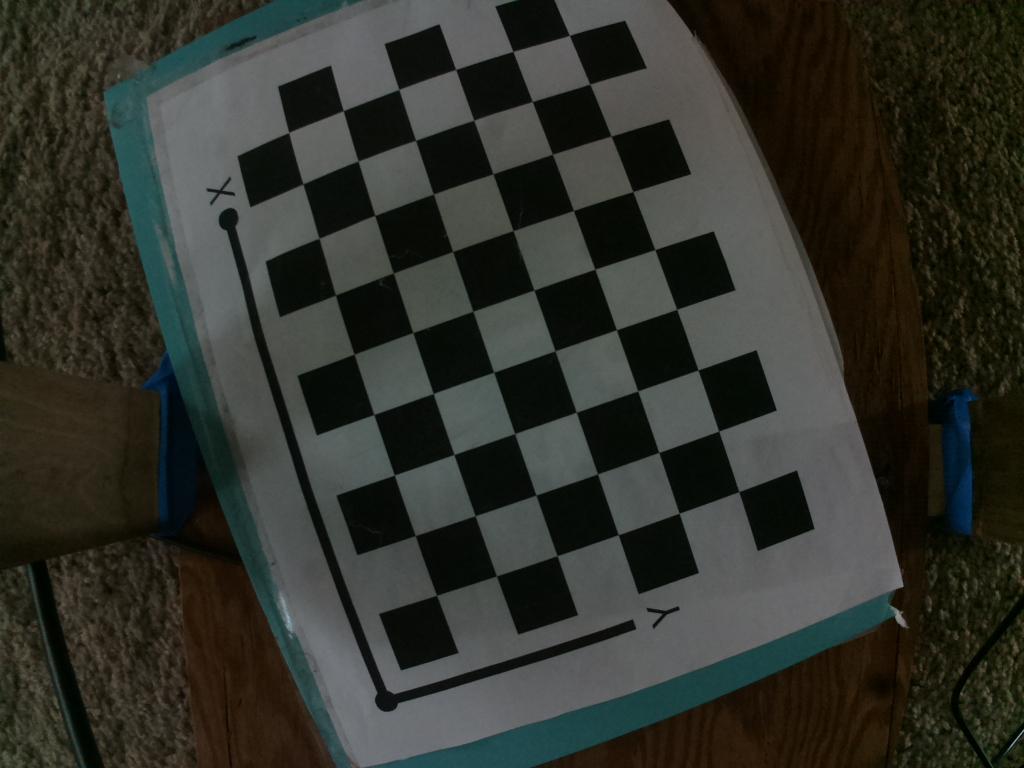

Supplement: Supplementary file 1 [file sensors-23-00560-s001.zip › Combined Data/Calibration/Calibration picam/9 (6).jpeg]

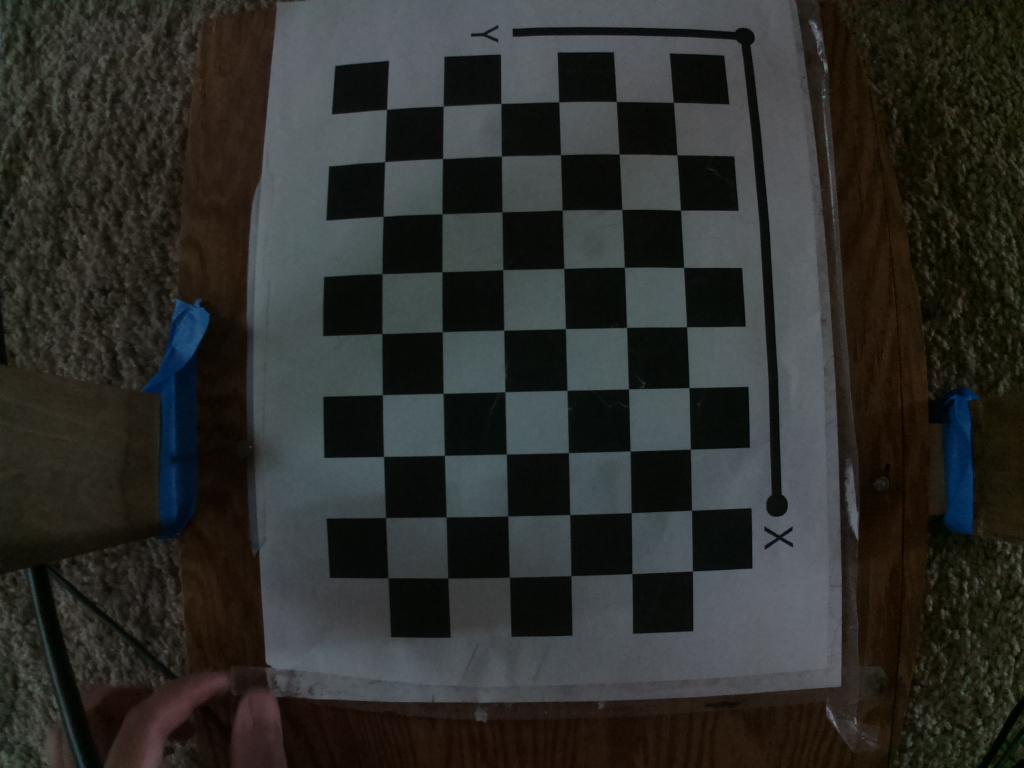

Supplement: Supplementary file 1 [file sensors-23-00560-s001.zip › Combined Data/Calibration/Calibration picam/9.jpeg]

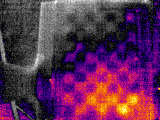

Supplement: Supplementary file 1 [file sensors-23-00560-s001.zip › Combined Data/Calibration/Calibration thermal/rgb47.png]

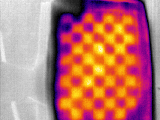

Supplement: Supplementary file 1 [file sensors-23-00560-s001.zip › Combined Data/Calibration/Calibration thermal/rgb48.png]

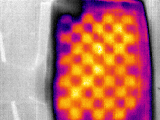

Supplement: Supplementary file 1 [file sensors-23-00560-s001.zip › Combined Data/Calibration/Calibration thermal/rgb49.png]

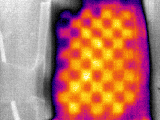

Supplement: Supplementary file 1 [file sensors-23-00560-s001.zip › Combined Data/Calibration/Calibration thermal/rgb50.png]

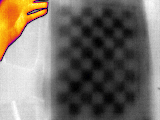

Supplement: Supplementary file 1 [file sensors-23-00560-s001.zip › Combined Data/Calibration/Calibration thermal/rgb51.png]

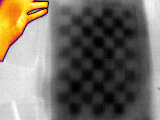

Supplement: Supplementary file 1 [file sensors-23-00560-s001.zip › Combined Data/Calibration/Calibration thermal/rgb52.png]
